# Comprehensive evaluation of primer pairs targeting the ammonia monooxygenase subunit A gene of complete ammonia-oxidizing *Nitrospira*

**DOI:** 10.1128/spectrum.00516-24

**Published:** 2024-08-21

**Authors:** Pieter Blom, Garrett J. Smith, Maartje A. H. J. van Kessel, Hanna Koch, Sebastian Lücker

**Affiliations:** 1 Department of Microbiology, Radboud Institute for Biological and Environmental Sciences, Radboud University, Nijmegen, The Netherlands; 2 Bioresources Unit, Center for Health & Bioresources, AIT Austrian Institute of Technology GmbH, Tulln an der Donau, Austria; University of Southern Denmark, Odense, Denmark

**Keywords:** comammox, primers, PCR, amplicon sequencing

## Abstract

Since the discovery of complete ammonia oxidizers (comammox) within the genus *Nitrospira*, their distribution and abundance across habitats have been intensively studied to better understand their ecological significance. Many primers targeting their ammonia monooxygenase subunit A gene (*amoA*) have been designed to detect and quantify comammox bacteria and to describe their community structure. We identified 38 published primers, but only few had high coverage and specificity for all known comammox *Nitrospira* or one of the two described subclades. For each target group, we comprehensively evaluated selected primer pairs using *in silico* analyses, endpoint PCRs, qPCRs, and amplicon sequencing on samples from various environments. Endpoint PCRs and qPCRs showed that the most commonly used primer pairs (comaA-244F/659R, comaB-244F/659R, and Ntsp-amoA162F/359R) produced several bands, which likely inflated quantifications via qPCR. In contrast, the recently published primer combinations CA377F/C576R, CB377F/C576R, and CA-CB377F/C576R resulted mostly in a single band. Furthermore, amplicon sequencing demonstrated that these primer combinations also captured the highest richness of comammox *Nitrospira*. Taken together, our results indicate that few existing comammox *amoA* primer combinations have both high specificity and coverage and that the choice of these high-specificity and high-coverage primer pairs substantially impacts the accurate detection, quantification, and community description of comammox bacteria. We, therefore, recommend using the CA377F/C576R, CB377F/C576R, and CA-CB377F/C576R primer pairs.

**IMPORTANCE:**

Bacteria that can fully convert ammonia via nitrite to nitrate, the complete ammonia oxidizers (comammox), were recently discovered and are found in many natural and engineered environments. PCR-based tools to study their abundance and diversity were rapidly developed, resulting in a plethora of primers available, many of which are widely used. The presence of comammox bacteria in an environment can, however, only be correctly determined if the used primers detect all members of this group while not detecting any other guilds. This study assesses the coverage and specificity of existing primers targeting comammox bacteria using both computational and standard molecular techniques, revealing large differences in their performance. The uniform usage of well-performing primers across studies could aid in generating comparable and generalizable data to better understand the importance of comammox bacteria in the environment.

## INTRODUCTION

The discovery of bacteria within the genus *Nitrospira* performing complete nitrification (1, 2) has changed our perception of this important nitrogen cycle process. These complete ammonia-oxidizing (comammox) bacteria are the third known microbial group besides ammonia-oxidizing archaea (AOA) and bacteria (AOB) to perform the first step of nitrification—the oxidation of ammonia to nitrite. However, they also catalyze the second step—nitrite oxidation to nitrate—that otherwise is performed by nitrite-oxidizing bacteria (NOB), such as the canonical members of the genus *Nitrospira*, the same genus comammox bacteria belong to. Complete nitrifiers thus defied the long-held paradigm of two-step nitrification with the division of metabolic labor between AOA or AOB and NOB (3). Although their existence has long been overlooked, their relevance for the biogeochemical nitrogen cycle has been confirmed over the past years as they have been ubiquitously identified in a broad range of natural (4) and engineered habitats (5).

Analogous to AOB and AOA, the detection, quantification, and community description of comammox *Nitrospira* are commonly performed using the functional marker gene *amoA*. This gene encodes subunit A of the ammonia monooxygenase, which is part of the larger gene family of copper-dependent membrane-bound monooxygenases (6). Based on phylogenetic analysis of *amoA* genes and corroborated by whole-genome phylogeny, comammox *Nitrospira* form two sister clades, clade A and clade B (1, 7, 8) that differ in their evolutionary history and potentially in their physiological properties as suggested by comparative genome analysis (9). In addition, further subdivisions within clade A (e.g., A.1 and A.2) and clade B have been proposed (10–12). In contrast to other nitrifying guilds, the 16S rRNA gene cannot be used to identify comammox species since they do not form a monophyletic group within the genus *Nitrospira* based on 16S rRNA phylogeny. Thus, primers and probes targeting the 16S rRNA gene cannot reliably differentiate comammox from canonical nitrite-oxidizing *Nitrospira* (13).

To accurately describe the nitrifying communities, many studies designed and published primers targeting the *amoA* of all comammox *Nitrospira* (14), the separate clades (7, 15), or specific phylotypes (16). Pioneering studies revealed severe differences in the performance of widely used primer pairs in similar habitats (17, 18). In addition, various studies have reported difficulties in obtaining specific PCR products when applying certain primer combinations (19). To avoid false-negative and false-positive detections in environmental studies, it is vital to verify that primer pairs have high coverage and specificity for their target group *in silico* and to confirm their specificity experimentally.

In this study, we comprehensively evaluated 38 published comammox *Nitrospira amoA*-targeted primer pairs by describing their *in silico* and experimental performance—similar to other studies focusing on other nitrogen-cycling genes (20–22). In the *in silico* analysis, the coverage and specificity of all primers were determined against a manually curated database containing genome-derived comammox *Nitrospira amoA* and other copper-dependent membrane-bound monooxygenase subunit A (Cu-*mmoA*) genes. Based on this analysis, selected primer pairs were tested for their specific and reliable amplification on a broad range of environmental samples using PCR, qPCR, and amplicon sequencing. Altogether, this approach resulted in the identification of the most suitable primer pairs to detect and quantify comammox *Nitrospira*.

## MATERIALS AND METHODS

### Generation of a Cu-*mmoA* gene sequence data set

Multiple strategies were applied to generate the Cu-*mmoA* gene data set used in this study. The genomes of isolates included in the IMG database (access date: 03 May 2022), noting that some metagenomic bins fall in this category, were surveyed for the presence of Pfam02461 for methane/ammonia monooxygenase subunit A, and these genes were downloaded. For additional non-comammox *amoA* sequences, a tBLASTn survey against the Refseq Genome Database (refseq_genomes; access date: 30 June 2022) was performed using phylogenetically diverse Cu-MMO protein sequences (AAC38651, WP_026223175, VUZ86048, WP_142864601, WP_011330982, BAE86885, RLA17056.1) as query. Using metascan (23), comammox *amoA* sequences were queried for and extracted from a data set containing 194 genomes identified as Nitrospiraceae by GTDB R207, 60 Nitrospiraceae genomes from the GEM catalog (24), 54 *Nitrospira* lineage I and lineage II genomes (deposited to NCBI GenBank between July 2021 and August 2022), and 6 additional *Nitrospira* genomes that failed the quality check of GTDB but were deposited as *Nitrospira* assembly on NCBI before July 2021. Moreover, other genome-derived comammox *amoA* sequences were included based on existing data sets (25, 26). All obtained Cu-*mmoA* gene sequences were filtered using a minimum sequence length of 700 bp, and identical sequences were removed. These 487 high-quality, non-redundant sequences were aligned using Muscle (27), and a phylogenetic tree was calculated using FastTree 2.1.5 (28) implemented in Geneious Prime 2022 (Dotmatics, Boston, MA, USA). The phylogenetic analysis of the sequences (6), including MBAE-like Cu-*mmoA* (29), was used to classify the sequences as 108 comammox *amoA* and 379 non-comammox Cu-*mmoA* gene sequences. No AOA *amoA* sequences were included in the analyses, but the amoA of *Nitrosotalea* sp. Nd2 was used as outgroup to root the phylogenetic tree. For phylogenetic analysis focusing on comammox bacteria, 108 comammox and 6 betaproteobacterial AOB *amoA* sequences of our reference data set were aligned using Muscle aligner with default settings implemented in Geneious Prime 2022, and a maximum likelihood phylogenetic tree was calculated using iq-tree v1.6.12 (30) with 1,000 ultrafast bootstrapping iterations on W-IQ-tree (31) using TIM3 + F + I + G4 identified as best fitting model by ModelFinder (32) integrated in W-IQ-tree. The tree was visualized and rooted using the AOB *amoA* sequences as outgroup in iTOL (33). The nucleotide sequences of the Cu-*mmoA* gene data set can be obtained from zenodo.org (https://doi.org/10.5281/zenodo.10251252).

### *In silico* primer evaluation

In total, 38 primers (Table S1) were analyzed by comparing them to the obtained reference sequence data set using CLC genomics workbench v.21.04 (Qiagen Sciences, Germantown, MD, USA). The tool “find binding sites and create fragments” was used with default settings, with ≥11 bp match to the target sequence required and a minimum of two consecutive matching base pairs at the 3′ end to the sequences. Subsequently, the coverage of each primer to clade A and clade B *amoA* sequences, AOB *amoA* sequences, and other Cu-*mmoA* sequences was calculated separately allowing ≤3 mismatches (MM). Since the primers CamoA_1F, Nino_amoA_19F, Nitrosa amoA-812R, Inopinata amoA-815R, Nitrificans amoA-836R, and CamoA_846R (Table S1) bind within the first or last 60 bp of the *amoA* gene (Fig. S1) and these regions are not well covered by the reference data set, a second reference sequence data set containing only full-length Cu-*mmoA* sequences was used for the evaluation of these primers. Here, 267 of the 486 sequences were included, all containing the start and stop codons when translated *in silico*. All other primers (*n* = 32) were queried against the data set containing 486 Cu-*mmoA* sequences.

For analysis of the three primer pairs with the highest overall coverage of their respective target group, the primer pairs were matched against the full reference data set allowing only hits that fulfilled the following criteria for both primers of the pair: minimum two consecutive base pairs in 3′ end, a maximum number of allowed MM ≤3, and the minimum matching base pairs set to 13 bp for comaA-244F/659R and comaB-244F/659R, 17 bp for Ntsp-amoA-162F/359R, and 15 bp for CA377F/C576R and CB377F/C576R. To analyze the distribution of MM in their target regions for selected primers, all comammox *amoA* sequences were aligned using the default settings of the Muscle aligner implemented in Geneious Prime 2022. The aligned sequences of the primer binding sites were extracted, and the aligned sequences were reverse complemented for reverse primer binding regions. The sequence logos of all binding regions were visualized as frequency plots using the webtool https://weblogo.berkeley.edu/logo.cgi with default settings.

### Sample collection and DNA extraction

Samples from 14 different ecosystems were collected from the following locations in The Netherlands in January 2022: biofilm from the anaerobic compartment of an in-house recirculating aquaculture system (RAS) (2); activated sludge from the municipal wastewater treatment plants (WWTPs) of Nijmegen (WN; N 51°50*'*56.3*"*, E 5°47*'*52.8*"*) and Arnhem (WA; N 51°57*'*59.0*"*, E 5°51*'*20.2*"*); top-layer sediment from a pond in a botanical garden (SeG; N 51°49*'*13.5*"*, E 5°52*'*27.3*"*); top-layer soil from a park (SoP; N 51°49*'*17.9*"*, E 5°52*'*12.3*"*), the Brakkenstein forest (SoF; N 51°49*'*12.7*"*, E 5°52*'*19.5*"*), and an agricultural field (SoA; N 51°50*'*39.4*"*, E 5°53*'*8.9*"*); and biomass from an in-house nitrifying membrane bioreactor (R1) (34) and a nitrifying continuous reactor (R2; unpublished data). Samples were kept on ice and immediately processed in the lab. Additional samples were collected previously and stored at −20°C: biofilm from canal walls in Amsterdam and Delft (CA and CD; 35), filter material from rapid sand filters in drinking water treatment plants (DWTPs) in Venray (DB) (25) and Sint Jansklooster (DS) (36), and sediment from the marine Lake Grevelingen (SeG) (37). DNA was extracted using the DNeasy PowerSoil Pro Kit (Qiagen, Germany) according to the manufacturer’s recommendations. The obtained DNA was quantified using the Qubit 2.0 fluorometer with the dsDNA HS Assay Kit (Invitrogen, USA).

### Endpoint PCR

PCRs were performed in a final volume of 25 µL containing 10 ng template DNA, 500 nM of each primer, and 1× PerfeCTa SYBR Green SuperMix (Quantabio, USA). An initial denaturation step (95°C, 5 min) was followed by 35 cycles (95°C, 30 s; 48/52/55°C, 30 s; 72°C, 30 s), followed by a final elongation step (10 min, 72°C) and cooling (15 min, 4°C). Primer details, including their respective annealing temperatures, are given in Table 1, and their sequences in Table S1. PCR products were examined for size and yield using gel electrophoresis (1.5% agarose, SB buffer, 80 V, 50 min; 0.1 µg/mL ethidium bromide).

**TABLE 1:** Primer pairs used in this study for endpoint PCR, qPCR, and amplicon sequencing

Forward primer(s)	Reverse primer	Target clade	Abbr.	Product length (bp)	*T*_*a*_ (°C)	Ref.
comaA-244F	comaA-659R	A	A.P	415	52	(8)
comaB-244F	comaB-659R	B	B.P	415		
Ntsp-amoA 162F	Ntsp-amoA 359R	A, B	AB.F	198	48	(14)
CA377F	C576R	A	A.J	235	55	(15)
CB377F	C576R	B	B.J	235		
CA377F/CB377F^*a*^	C576R	A, B	AB.J	235		

^*a*^ Abbreviated as CA-CB377F/C576R in the text.

### qPCR standard preparation

The standard for clade A comammox qPCR was prepared by cloning the *amoA* gene of “*Candidatus* Nitrospira kreftii” (34, 38) into a pGEM T Easy vector (Promega, Madison, USA). The *amoA* gene was amplified with the CamoA_1F/CamoA_846R primer pair (39) from “*Candidatus* Nitrospira kreftii” biomass as described under Endpoint PCR, purified with the GeneJET PCR Purification Kit (ThermoScientific, Waltham, USA), and ligated with a linear pGEM T Easy vector using T7 ligase (ThermoScientific). Plasmids were transformed into competent *E. coli* BL21 cells and isolated from transformants using GeneJET Plasmid Miniprep Kit (Thermofisher). The qPCR standard for clade B comammox was commercially synthesized (GeneUniversal, Delaware, USA) and contains the *amoA* gene of RSF3 (38) in a pUC19 vector. To produce linear qPCR standards, an endpoint PCR in triplicate with the M13F/M13R primer pair (40) was performed, and pooled PCR products were purified with the GeneJET PCR Purification Kit (ThermoScientific, Waltham, USA), quantified using the Qubit 2.0 fluorometer, and sequenced using Sanger sequencing (Baseclear, Leiden, The Netherlands). For preparing a qPCR standard series, consecutive 10-fold dilutions were prepared in DEPC-treated water to cover a range of 10^0^–10^8^ copies/µL.

### AmoA quantification by qPCR

All qPCRs were performed in triplicate in a C1000Touch Thermal Cycler, equipped with a CFX96 Real-Time System (Bio-Rad, Hercules, USA), and operated with Bio-Rad CFX Manager 3.0. PCR reactions were performed as described under Endpoint PCR, but using 40 cycles and followed by a melting curve ranging from 65°C to 95°C with 0.5°C increments of 5 s. One sample of each triplicate was checked for unspecific amplification by gel electrophoresis, as described under Endpoint PCR. All data were analyzed with Bio-Rad CFX Manager 3.0 (Bio-Rad), and the *Cq* was determined using single threshold. All qPCR statistics are reported in Table S2.

### Amplicon sequencing

Fragments for amplicon sequencing were obtained using a two-step PCR approach. In the first amplification step, triplicate reactions were performed (as described under Endpoint PCR, using 30 cycles and 50 µL reactions). After examination on gel, PCR products of the triplicate reactions were pooled and purified with the GeneJET PCR Purification Kit (ThermoScientific) and quantified using the Qubit 2.0 fluorometer. The second amplification step was performed in a single 50 µL reaction containing 25 ng purified PCR product. The same primers were used in the second PCR as in the first but modified with a sequencing tag at the 5′ end (forward primer tag: 5′-TCGTCGGCAGCGTCAGATGTGTATAAGAGACAG-3′; reverse primer tag: 5′-GTCTCGTGGGCTCGGAGATGTGTATAAGAGACA-3′). A slightly modified PCR program was used: initial denaturation (94°C, 4 min); seven cycles of denaturation (94°C, 30 s), annealing (48/52/55°C, 30 s), and elongation (72°C, 60 s); final elongation (72°C, 7 min). The PCR products were analyzed by gel electrophoresis (see Endpoint PCR) and purified as described under qPCR standard preparation. Tagged PCR products were paired-end sequenced on a MiSeq sequencer (Illumina, San Diego, USA) using Herculase II Fusion DNA Polymerase Nextera XT Index V2 Kit (Illumina) following the “16S Metagenomic Sequencing Library Preparation Part #10544223 Rev. B” protocol at Macrogen Europe (Amsterdam, The Netherlands). The total number of obtained reads was 4,379,874, with an average number of 218,994 ± 36,178 raw reads per sample.

### Amplicon sequence variant quantification and identification

Raw reads were processed using DADA2 v1.22.0 (41) in R (v4.1.2; R Core Team, 2022) with default parameters unless otherwise noted. For each primer combination separately, primer regions were removed by left trimming and truncating the read to the expected length (Table S3). Primer-trimmed reads were additionally filtered for contamination (settings: phiX removal, ID matching, maxE 1) followed by denoising and dereplication (settings: error learning bases 1e10, pooling during denoising, overhang trimming during merging, minimum overlap depending on the primer combination), amplicon sequence variant (ASV) calling, and read abundance counting. Due to their longer amplicon length, reads sequenced from comaA-244F/659R amplicons were additionally filtered by length (minimum length: 200 bps). On average, 75% of reads and 35% of bases were retained for primers Ntsp-amoA-162F/359R, 85% and 54% for CA-CB377F/C576R, and 36% and 32% for comaA-244F/659R primers. ASVs from all data sets were compared to the reference data set using command line BLASTN (version, 2.13.0+) for taxonomic affiliation to their highest bitscore match with ≥75% nucleotide identity and alignments ≥66% of the expected amplicon length. Any ASV aligning <33% of its expected length was considered unaligned. ASV read counts were additionally aggregated to their best matching genomic reference with a minimum of 90% nucleotide identity over at least 66% of the expected amplicon length.

For phylogenetic placement of the obtained comammox ASVs, 108 comammox *amoA* sequences, 6 AOB *amoA* sequences, and the ASV sequences from each primer pair separately were aligned using muscle implemented in Geneious Prime 2022. For each nucleotide alignment, the model TIM3 + F + I + G4 was identified as the best fitting model using the model finder of W-IQ-tree. A reference *amoA* tree without ASV sequences was calculated using RAxML-NG v1.1.0 (42) implemented in the CIPRES science gateway v3.3 using the model TIM3 + F + I + G4 and 500 bootstrap iterations. Then, ASVs of every primer pair were placed separately onto the topology of this reference tree using EPA-NG v.0.3.8 (43) implemented in the CIPRES platform with the following settings: “filter-max: 7; filter-min: 1; precision: 10; dyn-heur: 0.99999 –preserve-rooting: off.” The phylogenetic placement of the ASVs onto the *amoA* phylogenetic tree was visualized in iTOL (33).

Further data processing, analyses, and visualization were performed in R (v4) primarily using the tidyverse and ggtree packages.

## RESULTS

### *In silico* coverage and specificity of published comammox primers

**Fig 1:**
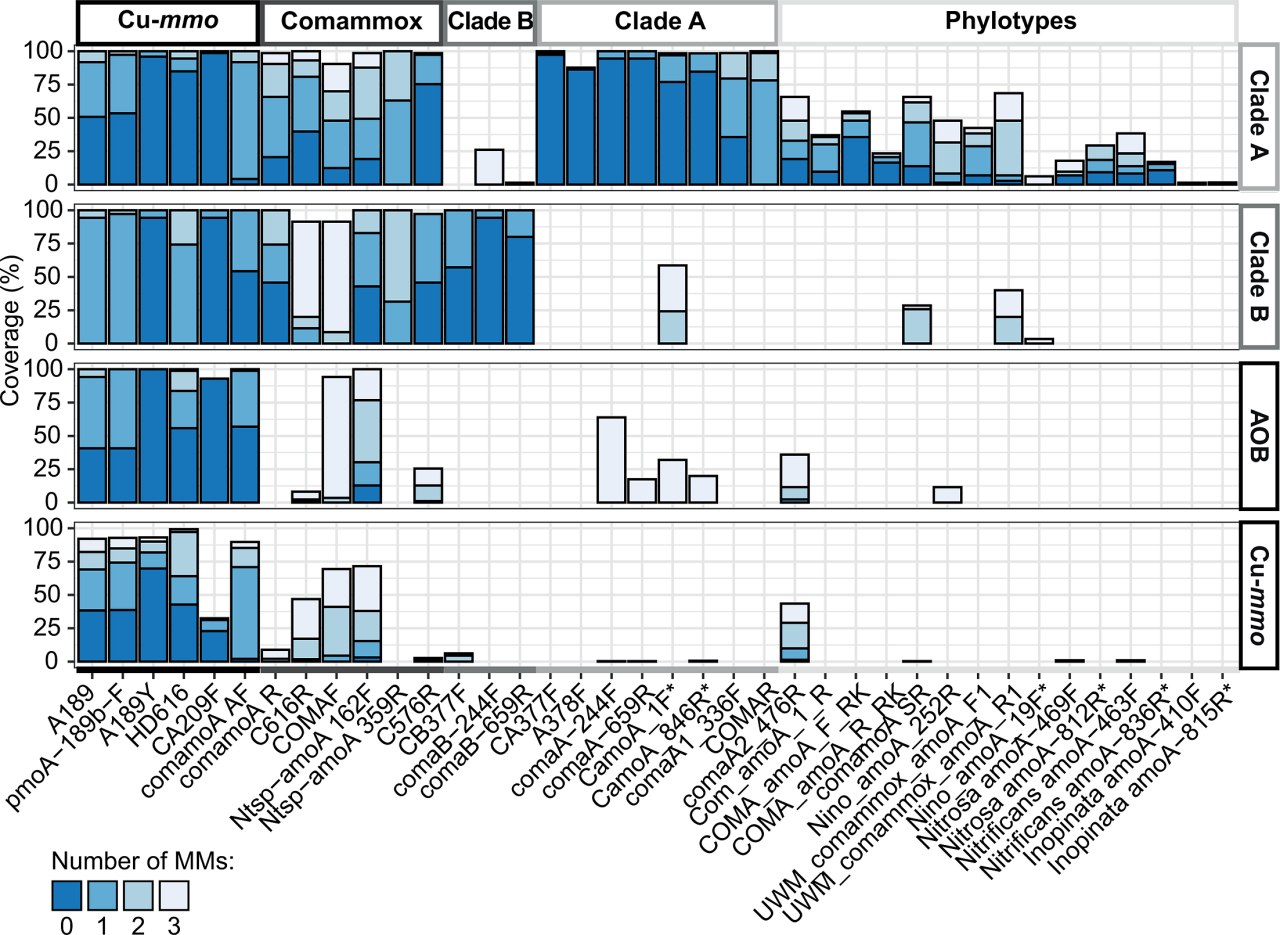
Coverage and specificity of comammox *Nitrospira amoA*-targeted primers. The percentage of target sequences per group each primer binds to with ≤3 mismatches and without MM at the last two positions of the 3′ end (i.e., coverage) is shown for comammox clade A and clade B, AOB *amoA* sequences, and other Cu-*mmoA* sequences. Based on their coverage of each of these clades, primers were categorized as general Cu-*mmoA*, general comammox, clade A-specific, clade B-specific, or phylotype-specific primers. Primers analyzed using a smaller data set comprising full-length sequences were marked with an asterisk. See Table S1 for primer details and Fig. S2 for a more detailed representation of Cu-*mmoA* group coverages.

Many studies designed and applied PCR-based methods to assess the diversity and abundance of comammox *Nitrospira* to gain insights into their ecological importance in different environments. In this study, 38 published primers targeting comammox *amoA* sequences were evaluated *in silico* (Table S1; Fig. S1). Coverage and specificity were determined against a manually curated database of comammox *amoA* and other Cu-*mmoA* gene sequences. The analyzed primers targeted either the whole Cu-*mmoA* family, all comammox *amoA*, comammox clade A or clade B specifically, or certain comammox *Nitrospira* phylotypes (Fig. 1; Fig. S2).

Several primers targeting the whole Cu-*mmoA* family are available, including different modifications of the forward primer A189 (44, 45) and the highly degenerated reverse primer HD616 (12, 45), but also comamoA AF (46) and CA209f (12) target a large proportion of Cu-*mmoA* genes with ≤3 MM (Fig. S2). According to our analyses, few primers are available that target both comammox clades with high coverage and specificity with ≤3 MM, most of which are reverse primers (Ntsp-amoA 359R, C576R, comamoA R, 616R) (12, 14, 47). Contrastingly, the published general comammox *amoA* forward primers (COMAF, Ntsp-amoA 162F) (14, 48) also target many additional Cu-*mmoA* genes and, surprisingly, also have ≥1 MM to most comammox *amoA* sequences (Fig. 1). Our analyses found that primer C576r has the highest coverage and specificity, while all other general comammox primers target >90% of all comammox sequences only when allowing ≥2 MM. Consequently, while no high-coverage, high-specificity primer pair targeting all comammox bacteria exists, the existing reverse primers can be combined with the general Cu-*mmoA* forward primers, as has, for instance, been done for comamoA R and comamoA F (47) or C576r and CA206f (12).

For clade A comammox bacteria, CA377F (15) and the primer pairs comaA-244F/659R (7) and CamoA_1F/846R (39) are highly specific and have a high group coverage (>90%). Notably, the PCR product of the latter primer pair is too long for amplicon sequencing and qPCR. Primer A378f (12) also has excellent specificity but targets <90% of clade A sequences (Fig. 1). Contrastingly, the primers comaA1_336F (49) and COMAR (48) only target >90% of all clade A comammox *amoA* sequences when allowing ≥2 MM. For clade B comammox *amoA* sequences, the only primers that have high coverage and specificity are CB377F (15) and the primer pair comaB-244F/659R (7). All other evaluated primers cover only a subset of comammox *amoA* sequences since they were mostly designed to target specific comammox phylotypes.

**Fig 2:**
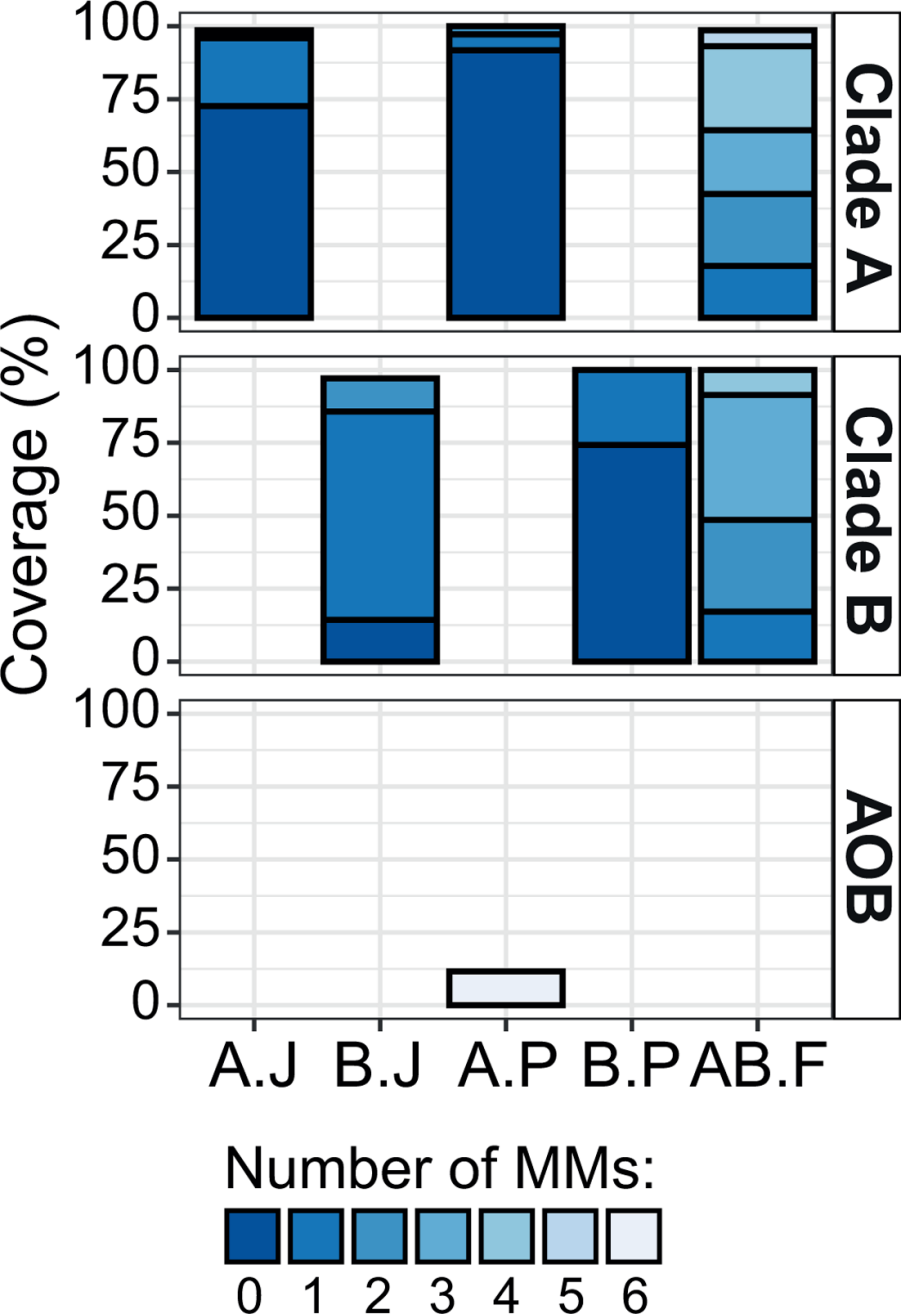
Coverage and specificity for selected comammox *Nitrospira amoA*-targeted primer pairs. The percentage of target (clade A and clade B) and non-target (AOB) *amoA* sequences that the primer pairs bind to (i.e., coverage) is shown. The maximum number of mismatches per primer was 3 without MM at the last two positions of the 3′ end. The selected comammox *amoA* primer pairs are CA377F/C576R (A.J), CB377F/C576R (B.J), comaA244F/659R (A.P), comaB244F/659R (B.P), and Ntsp-amoA162F/359R (AB.F).

In summary, our *in silico* analysis identified the clade-specific primer pairs CA377F/C576R and CB377F/C576R (15), and comaA-244F/659R and comaB-244F/659R (7) as the primers with the highest coverage (Fig. 2; Fig. S3) and highest specificity for their target group (Fig. 1; Fig. S2). In contrast, the widely used primer pair Ntsp-amoA 162F/359R (14) was found to have ≥1 MM to all target sequences (Fig. 2; Fig. S3).

### Endpoint PCR performance of high-specificity and high-coverage primers

Based on the *in silico* analysis, we selected the primer combinations with the highest coverage and specificity to test in endpoint PCRs using 14 samples that cover a wide range of engineered and natural ecosystems. For each comammox clade, two primer pairs were selected: CA377F/C576R (expected amplicon size 235 bp) (15) and comaA-244F/659R (415 bp) (7) for clade A, and CB377F/C576R (235 bp) (15) and comaB-244F/659R (415 bp) (7) for clade B. However, according to our analysis, no high-coverage, high-specificity primer pair targeting all comammox bacteria is available since the widely used Ntsp-amoA-162F/359R (14) did not fulfill our criteria (Fig. 1 and 2). Thus, we tested the use of an equimolar mixture of CA377F and CB377F as forward primer in combination with C576R (235 bp), which then was compared to the performance of the Ntsp-amoA162F/359R primer pair (198 bp) (14).

For the comammox clade A-specific PCRs, products of the expected size were obtained for most samples using the primer combinations CA377F/C576R and comaA-244F/659R (Fig. S4). However, primers comaA-244F/659R showed unspecific amplification for some samples to a much larger extent than CA377F/C576R, especially in samples where the latter primer combination yielded little to no product. In the comammox clade B-specific PCRs, stark differences were observed between the primer pairs and tested samples. PCR products of the expected size were obtained for a subset of samples using CB377F/C576R, while for the comaB-244F/659R primer pair, unspecific PCR amplification was observed for all samples (Fig. S4). However, also CB377F/C576R yielded unspecific PCR products for some samples but to a much lesser extent. The general comammox primer combinations tested, i.e., those targeting both comammox clades, yielded comparable results for most samples (Fig. S4). As expected, the amplification pattern for the samples tested with the combination CA-CB377F/C576R largely reflected the clade-specific PCRs but surprisingly appeared to be slightly more specific than the individual primer pairs. In contrast, primers Ntsp-amoA162F/359R, while yielding an amplicon of the expected size for most samples that contained comammox bacteria according to the other PCRs performed, also showed a large degree of unspecific amplification.

In conclusion, the comammox clade-specific forward primers CA377F and CB377F together with the general reverse primer C576R appear to be the best available option to screen for the presence of comammox *Nitrospira*. They can be used to either detect clade A and B comammox bacteria separately or be combined and used as general comammox primer combination.

### Quantitative PCR reveals systematic biases of primer pairs

**Fig 3:**
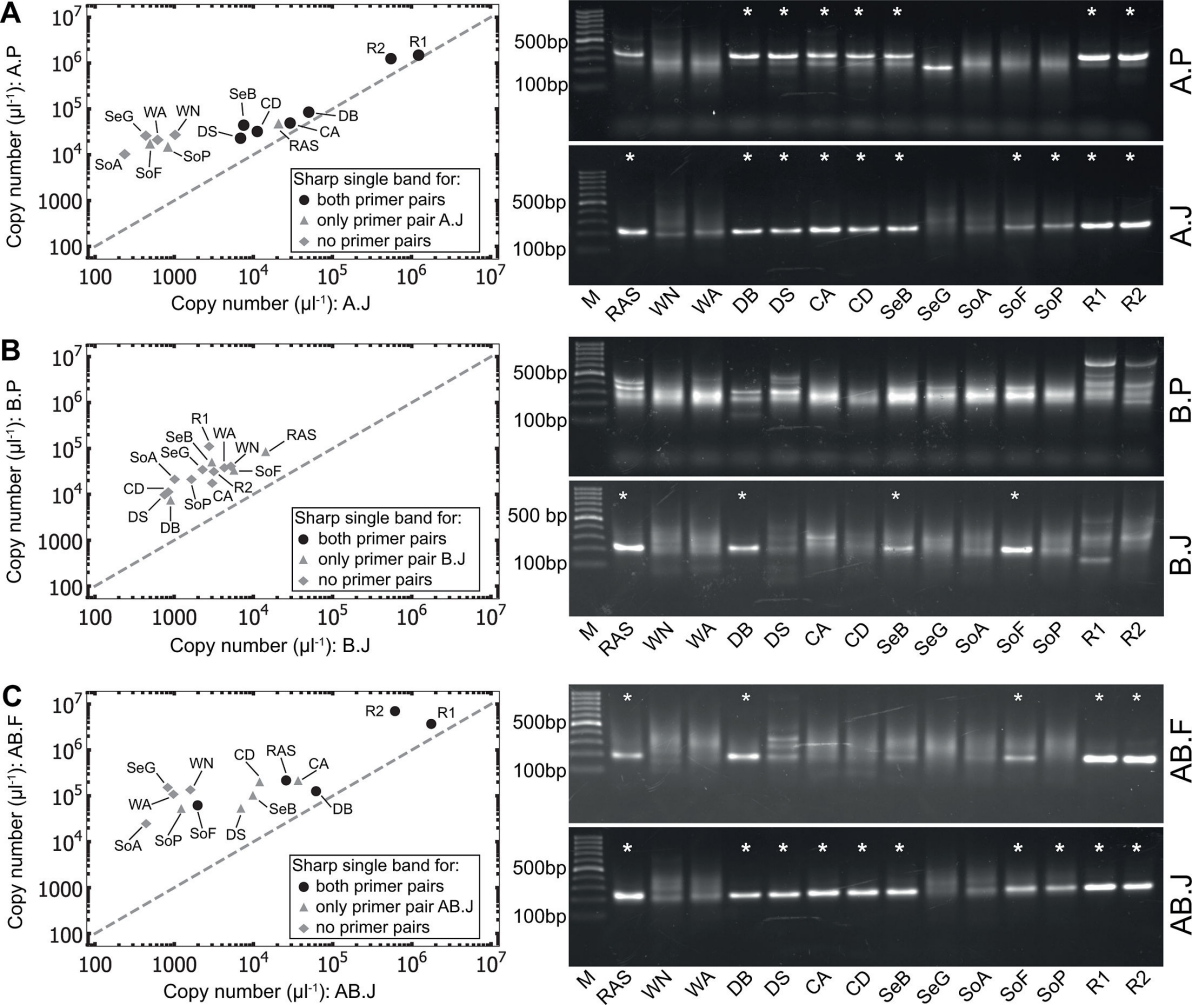
Comparison of copy numbers and amplification products for primer pairs with the same target group. (Left) Comparing detected copy numbers for (**A**) CA377F/C576R (A.J) with comaA-244F/659R (A.P); (**B**) CB377F/C576R (B.J) with comaB-244F/659R (B.P); (**C**) CA-CB377F/C576R (AB.J) with Ntsp-amoA162F/359R (AB.F); black circles indicate a sharp band for both primer pairs, gray triangles indicate a sharp band for the primer pair shown on the x-axis, and gray diamonds indicate lack of sharp bands for both primer pairs. (Right) Visualization of the obtained qPCR products by agarose gel electrophoresis. Defined single bands are indicated with asterisks. A 100-bp marker (M) was used for size estimation. DNA extracted from the following habitats was used: a recirculating aquaculture system biofilm; two samples of activated sludge from two different WWTPs (WA; WN); two rapid sand filter samples from different drinking water treatment plants (DB; DS); two biofilm samples from canals in two Dutch cities (CA; CD); sediment samples from both freshwater (SeB) and brackish systems (SeG); soil samples from a cropland (SoA), a forest (SoF), and a park (SoP); and biomass from two nitrifying bioreactors (R1; R2).

We performed qPCRs for the primer combinations tested by endpoint PCR using DNA obtained from the same 14 ecosystems, and the results largely corroborated the observations of the endpoint PCRs (Fig. 3). In qPCR assays targeting comammox clade A, the primer pair comaA-244F/659R consistently detected higher *amoA* copy numbers than CA377F/C576R. A similar trend was observed for the general comammox primer combinations tested, where the primers Ntsp-amoA162F/Ntsp-amoA359R invariably yielded higher copy numbers than CA-CB377F/C576R, indicating systematic biases of these primer pairs. For clade B comammox *Nitrospira,* such a comparison was not possible since no specific PCR product was obtained using the primer pair comaB-244F/659R, similar to the endpoint PCR results. Therefore, to validate the performance of the CA-CB377F/C576R combination, the results were benchmarked against the sum of *amoA* copy numbers obtained using the separate primer pairs CA377F/C576R and CB377F/C576R, which yielded comparable numbers (Fig. S5).

After the last PCR cycle, melting curve analysis is commonly performed in qPCRs to verify the specificity of amplification (50), indicated by a single sharp peak at the same temperature as for the standard DNA. Here, melting curve analyses generally supported the gel electrophoresis results (Fig. 3), but a few discrepancies were observed (Fig. S6). For instance, two distinct peaks or maxima at temperatures differing from the standards were observed for the primer pairs comaA-244F/659R, CA377F/C576R, and CA-CB377F/C576R. Furthermore, the melting curve analyses for comaA-244F/659R and especially comaB-244F/659R failed to reveal off-target amplification, yielding identical sharp melting curve peaks as the standard DNA, while gel electrophoresis clearly showed unspecific amplification. These results suggest that the combination of melting curve analysis and visual inspection of obtained PCR products by gel electrophoresis is essential for the identification of false-positive amplification and overestimation of comammox *Nitrospira amoA* genes in qPCR-based analyses.

### Amplicon sequencing of selected samples reveals amplification biases

PCR amplification yielded specific PCR products for only four samples (SeB, SoF, RAS, and DB) with all tested primer combinations, except for comaB-244F/659R. Therefore, amplicon sequencing was performed only on these four samples using five primer sets (CA377F/C576R, comaA-244F/659R, CB377F/C576R, CA-CB377F/C576R, and Ntsp-amoA162F/359R). Across all samples and primer combinations, a total of 3,874 ASVs were obtained. There was no apparent relationship between sequencing depth and ASV recovery. While comaA-244F/659R yielded among the lowest number of reads and ASV counts, the Ntsp-amoA-162F/359R primer pair recovered the largest number of ASVs despite having one of the lowest read counts. Sufficient sequencing depth was obtained for all samples and each primer pair, as indicated by rarefaction curves (Fig. S7).

A majority of ASVs and reads could be assigned to comammox *amoA*. A total of 1,337 and 424 ASVs were affiliated with comammox clade A and clade B *amoA*, respectively, which combined accounted for 83%–100% of reads (93.2% on average), except comaA-244F/659R in one sample (only 44.9% of reads for SoF). However, despite the use of comammox *amoA*-specific primers, 2,092 ASVs could not be affiliated with any Cu-*mmoA*. These unknown ASVs on average accounted for 6.7% of the obtained reads but constituted a significantly higher proportion in some cases (55.1% in SoF for comaA-244F/comaA-659R). Additionally, 11 ASVs were affiliated with the canonical AOB *Nitrosomonas*, *Nitrosospira*, and *Nitrosovibrio* (Table S4; Fig. S8), accounting for a maximum of 0.1% of total reads. In general, off-target amplification varied by both ecosystem and primer combination, and no combination amplified solely comammox *amoA* genes. Thus, while the number of off-target ASVs could be substantial, they typically constituted a small fraction of reads compared to comammox ASVs.

**Fig 4:**
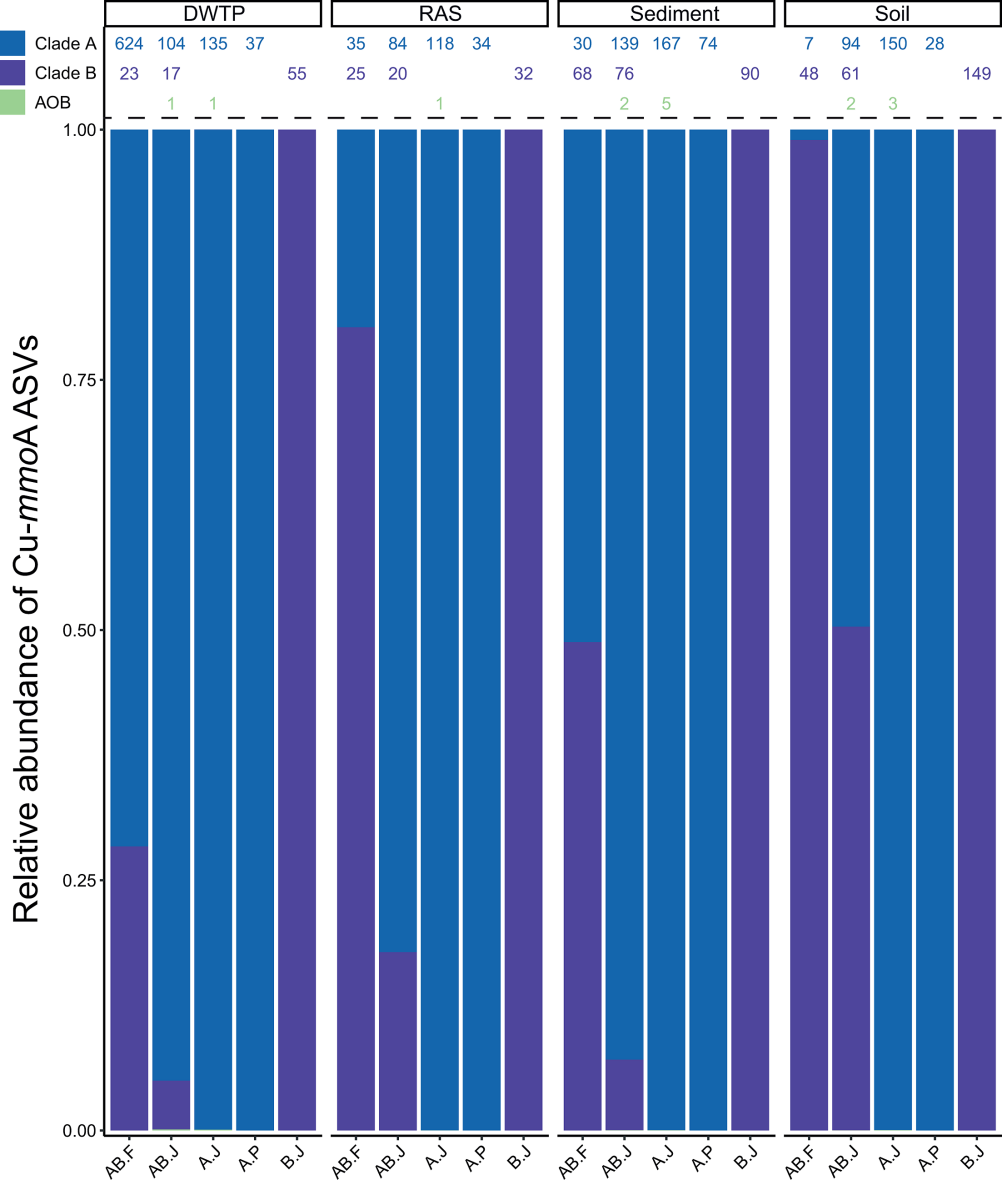
Relative abundance and absolute counts of ASVs obtained with selected *amoA*-targeted primer pairs. (Bottom) The relative abundance of comammox clade A and clade B, and AOB *amoA* ASVs as well as (top) the number of unique ASVs per group obtained by amplicon sequencing using the primer pairs Ntsp-amoA162/359R (AB.F), CA-CB377F/C576R (AB.J), CA377F/C576R (A.J), comaA- 244F/659R (A.P), and CB377F/C576R (B.J) on four selected habitats: DWTP (DB), RAS, sediment (SeB), and soil (SoF).

The different primer combinations recovered vastly different comammox community structures. Primers Ntsp-amoA162F/A359R yielded 770 comammox ASVs, two- to fourfold more than the other primer pairs (121–334 ASVs). This large number was primarily driven by the ASV yield from one sample (DB) that constituted 647 comammox ASVs. All clade-specific primer sets solely obtained ASVs belonging to the respective clade (Fig. 4). The primer pairs CA377F/C576R and CB377F/C576R separately led to the generation of more ASVs (319 and 217 ASVs, respectively) than the use of the primer combination CA-CB377F/C576R (334 ASVs), suggesting that mixing the forward primers reduces recovery of sequence diversity. Even if highly variable between samples, there was a consistent difference in the ratio of relative abundances between clade A and clade B for the primer combinations targeting both comammox clades. While CA-CB377F/C576R recovered a clade A:B ratio of on average 4.00, this was 0.57 for Ntsp-amoA162F/359R (Fig. 4). We note that the ratios of clade A:B obtained with CA-CB377F/C576R by amplicon sequencing differ from the ratios of the clade abundances determined by qPCR using primer pairs CA377F/C576R and CB377F/C576R separately (Fig. S9). This finding further highlights apparent discrepancies arising from mixing the forward primers to assess both comammox clades simultaneously. Furthermore, the differences in observed population structure between the primer pairs show that the choice of primer combination has a major impact on the results obtained, especially the observed differences in clade A:B ratio might indicate incomplete coverage of the comammox species present in either one or both clades.

**Fig 5:**
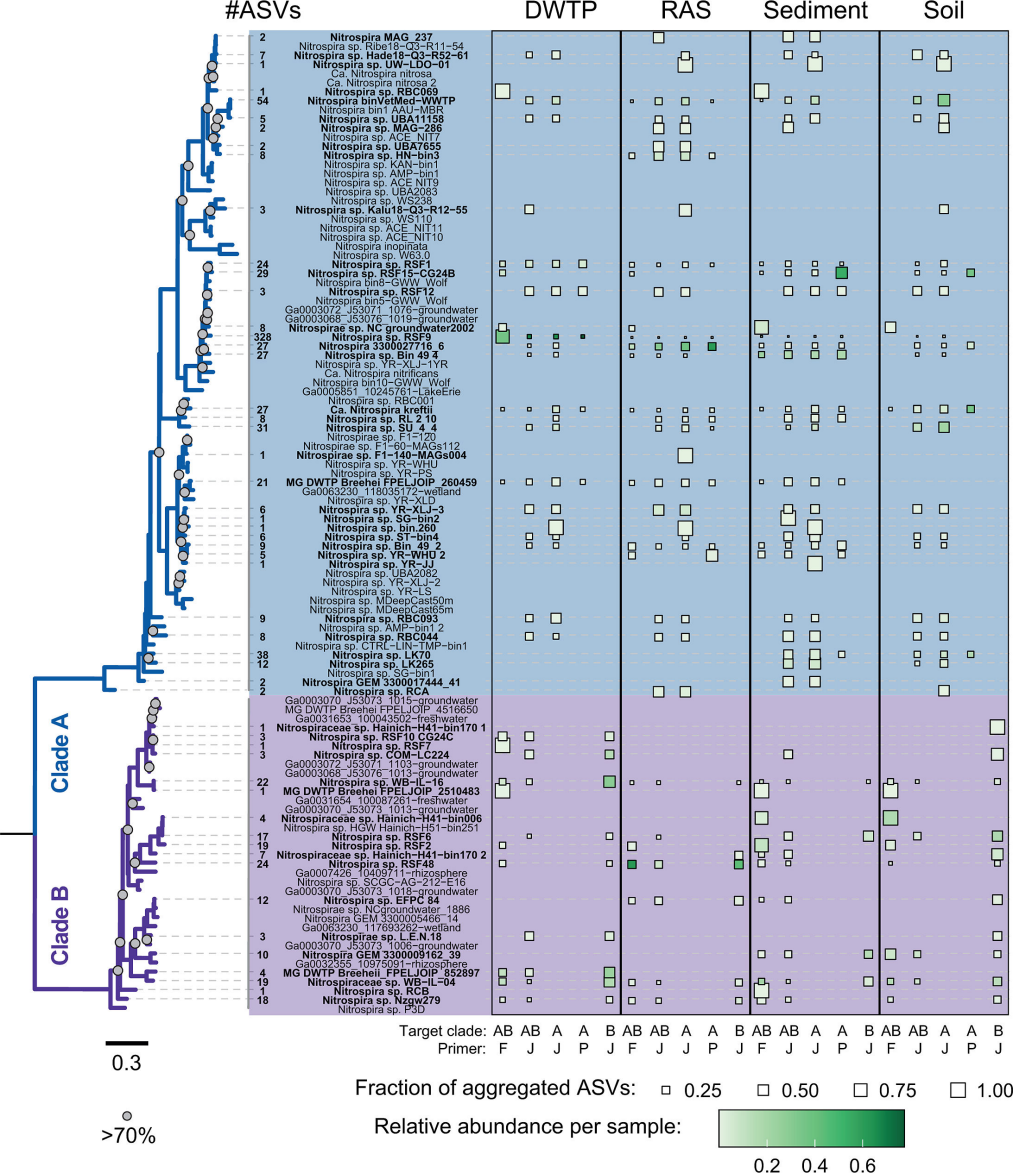
Phylogenetic diversity of ASVs matching to genome-derived *amoA* sequences. All obtained comammox *amoA* ASVs were aggregated to their closest reference sequence extracted from available comammox genomes and MAGs, with a cutoff threshold of >90%. The total number of aggregated ASVs is given for each reference to the left of the tip label. All ASVs are shown for habitats DWTP (DB), RAS, sediment (SeB), and soil (SoF) and primer pairs Ntsp-amoA162/359R (AB.F), CA-CB377F/C576R (AB.J), CA377F/C576R (A.J), comaA- 244F/659R (A.P), and CB377F/C576R (B.J). The square size represents the fraction of all ASVs aggregated to the respective genomic reference sequence present in the sample, the color intensity indicates the summed relative abundance of these ASVs in the data set. Gray circles on nodes in the phylogenetic tree represent >70% bootstrap support.

To explore the coverage of the distinct taxa within both comammox *Nitrospira* clades, ASVs and their corresponding reads were aggregated to their most similar genomic reference sequence, with a nucleotide identity cutoff >90%. At this threshold, some references accrued up to 328 ASVs across all primer pairs and ecosystems (Fig. 5; Fig. S10), but 562 of the 1,811 comammox ASVs were not matched to any reference sequence; notably, most of these unmatched ASVs came from the DWTP sample (DB) and mainly from the primer pair Ntsp-amoA162F/359R. Several comammox *amoA* reference sequences were recovered by nearly all primer sets and in essentially all tested ecosystems, such as, e.g., *Nitrospira* sp. RSF1, *Nitrospira* sp. RSF9, *Nitrospira* binVetMed-WWTP, and *Ca*. Nitrospira kreftii-like *amoA* genes. Many genomic references present in the database were not recovered, presumably due to their absence in these ecosystems.

Lastly, some taxa were not retrieved evenly from the same ecosystem using different primer sets (Fig. 5; Fig. S11), which impacted the detection of certain taxa and their observed relative abundances. In general, the primer pairs CA377F/C576R and CB377F/C576R, when used separately or with combined forward primers, recovered the largest diversity of clade A representatives compared to comaA-244F/659R and Ntsp-amoA162F/359R. However, the use of the primer combination CA-CB377F/C576R, while largely recovering similar taxa at similar relative abundances, apparently leads to an undersampling of clade B compared to the separate clade-specific primer pairs (especially in samples SeB and SoF).

## DISCUSSION

In this study, we compared the performance of available comammox *amoA* primers to detect and quantify comammox *Nitrospira* as well as describe their community composition, which is crucial for understanding their ecophysiological role in various environments. Despite the prediction of comparably high coverage and specificity in the *in silico* analyses, endpoint and qPCRs revealed pronounced differences for both the clade-specific and general primer combinations tested. Previously, multiple studies reported non-specific product formation for several comammox *Nitrospira amoA*-specific primer pairs (16, 18, 49, 51, 52). Our results corroborate these findings, as unspecific PCR products were obtained for several commonly used primer combinations (i.e., Ntsp-amoA162F/359R, comaA-244/F659R, comaB-244F/659R; Fig. S4). These unspecific amplifications likely result in an overestimation of comammox *Nitrospira* present in a sample, as exemplified by the differences in obtained *amoA* copy numbers in our qPCR analyses (Fig. 3) and also indicated by the large number of unaligned ASVs in the amplicon sequencing data set (Fig. S8). In contrast, the primer pairs CA377F/C576R and CB377F/C576R (15) showed limited unspecific product formation, likely resulting in a more accurate estimation of absolute comammox abundances.

### Primer choice influences observed diversity

The selection of primers does not only affect the reliability of detection and quantification of comammox *Nitrospira*, but it also influences the observed community composition. Apparent biases in the detection of certain comammox clades and subclades have been reported previously for the clade-specific primer sets comaA-244F/659R and comaB-244F/659R as well as for the general primer pair Ntsp-amoA162F/359R (17, 18). For instance, in a full-scale groundwater treatment bioreactor system, Ntsp-amoA162F/359R overestimated the relative abundance of a clade A.2 ASV compared to a clade A.1 ASV, assumedly due to the higher number of MMs to the *amoA* sequence of the clade A.1 species (53). Similarly, while the primers CA377F/C576R were able to amplify clade A.1 operational taxonomic units (OTUs), these were not detected by the general comammox primers Ntsp-amoA162F/359R and comamoA F/R in soil samples, and both clade-specific primer pairs CA377F/C576R and CB377F/C576R generally performed better at detecting comammox species of low relative abundance and obtained higher numbers of OTUs compared to the general primer sets (17). Moreover, an underestimation of clade A over clade B by amplicon sequencing using primers Ntsp-amoA162F/359R compared to metagenomic analysis was observed in two of three analyzed wetland soil samples (18). Although community composition was not analyzed using metagenomics in this study, our results showed a similar trend as these previous reports, indicating a potential underestimation of clade A for the often used primer pair Ntsp-amoA162F/359R, likely caused by the low detection rate of certain clade A phylotypes due to the presence of several MMs between primer and target sequences.

### Primer recommendation amplicon studies

Thus, our results corroborate previous reports that CA377F/C576R and CB377F/C576R are the best performing clade-specific primer pairs currently available. Furthermore, we found the primer combination CA-CB377F/C576R to perform best for the simultaneous detection of all comammox *Nitrospira* as they showed the least unspecific product formation in endpoint PCRs and recovered a broader diversity in *amoA* amplicon sequencing analyses than other frequently used primers. Due to the amplicon length of 235 bp, these primers are well suited for qPCR as well as current short-read high-throughput amplicon sequencing methods (e.g., Illumina HiSeq). However, it should be noted that also these primers amplify some non-comammox *amoA* sequences (15), and the combination CA-CB377F/C576R appears to yield slightly fewer ASVs than the primer pairs CA377F/C576R and CB377F/C576R used separately. However, in qPCR assays, we obtained comparable absolute abundances using the combined primers as with the clade-specific primer pairs summed up. It was previously reported that dimer formation of CB377F/C576R hampers the sensitivity of clade B-specific qPCRs (15). Here, we did not observe dimer formation, but the PCR efficiency with primers CB377F/C576R still was the lowest of all qPCR assays. Our sequence analysis showed that this primer pair has MMs at positions 13 and 4 of CB377F and C576R, respectively, to several clade B sequences, and we, therefore, tried to optimize both primer sequences. However, replacing either or both primer positions by the degenerated base N increased the amount of unspecific amplification (data not shown), and we thus suggest the standardized usage of the primer sets CA377F/C576R, CB377F/C576R, and CA-CB377F/C576R for detecting clade A and B, or all comammox *Nitrospira*, respectively. Additionally, we note that CamoA_1F/846R is a high-specificity, high-coverage primer pair targeting clade A *amoA* that was not further evaluated in our qPCR and amplicon sequencing analyses due to the expected fragment length of 846 bp (39). This primer pair amplifies full-length clade A *amoA* sequences, which are invaluable for comprehensive phylogenetic analysis in addition to MAG-derived sequences.

### General recommendations to increase comparability of PCR-based studies

Besides the uniform usage of primers, other measures would also increase the comparability of studies applying *amoA*-targeting and especially qPCR-based approaches. Since melting curve analyses are difficult to interpret due to wide melting temperature ranges (Fig. S6), presumably caused by pronounced differences in GC content of *amoA* amplicons (16), we recommend implementing a specificity control into the standard *amoA* qPCR workflow by visualizing the PCR products by gel electrophoresis. Furthermore, a standardized bioinformatic workflow for analyzing *amoA* amplicons would increase comparability between studies. For example, clustering into OTUs is common practice for *amoA* as well as other marker gene amplicon studies, but the thresholds vary from 90% (54) to 97% (12) nucleotide identity, which may artificially aggregate distinct strains or ecotypes. The arbitrary definition of similarity thresholds for clustering can be avoided by using amplicon sequence variants, which are now commonly integrated into 16S rRNA gene amplicon analyses (41, 55). Implementation of approaches capturing each unique ASV maximizes the precision of analyses. In addition, read or gene identification by comparison to easily accessible but uncurated databases may discard novel variants or wrongly assign divergent sequences. Here, we suggest maximizing the accuracy of assignments using a genome-derived database of both on- and likely off-target reference genes.

### Conclusions

The described difficulties when using comammox *amoA* primer pairs have prompted researchers to design primers targeting the *amoB* (19), which have successfully been applied in several engineered systems (56, 57). However, these only target clade A comammox *Nitrospira,* and the high level of sequence divergence of the available clade B *amoB* genes makes it challenging to design primers targeting this clade. An alternative approach to increase specificity and sensitivity, especially in diverse habitats containing low abundances of comammox *Nitrospira*, is nested PCR. This approach has been successfully employed for amplicon sequencing by successively employing two different general comammox primer sets: first, the universal Cu-*mmoA* forward primer A189Y in combination with the general comammox reverse primer C576R followed by the general comammox-specific primer pair CA209F/C576R (12). However, while this approach can be useful for a general description of community compositions, it might increase PCR biases due to preferential amplification and thus might poorly reflect the structure of the analyzed community (58). Since the nested PCR approach cannot be used for quantification via qPCR, we tested the primer pair CA209F/C576R directly for PCR but observed highly unspecific amplification as indicated by excessive smearing in gel electrophoresis (data not shown).

In conclusion, in this study, we compared the coverage and specificity of 38 published comammox primers. This *in silico* analysis combined with experimental evaluation indicated that the selection of primers may have profound implications for describing the abundance and community composition when studying comammox *Nitrospira* in complex environmental samples. Based on our findings, we recommend the usage of CA377F/C576R for clade A, CB377F/C576R for clade B, and CA-CB377F/C576R for all comammox, but note that a re-evaluation of these primer pairs might be necessary in the future when the number of available *amoA* sequences, especially from comammox-containing metagenomes, has further increased.

## Data Availability

All raw sequence data and corresponding metadata have been deposited in the sequence read archive (SRA) database of NCBI under BioProject PRJNA977628.

## SUPPLEMENTAL MATERIAL

Supplemental material spectrum.00516-24-s0001.pdf Fig. S1 to S11; Tables S2 to S4

Table S1 spectrum.00516-24-s0002.xlsx Overview of all primer characteristics.

ASM does not own the copyrights to Supplemental Material that may be linked to, or accessed through, an article. The authors have granted ASM a non-exclusive, world-wide license to publish the Supplemental Material files. Please contact the corresponding author directly for reuse.
