# Supplementary material for: Comprehensive evaluation of primer pairs targeting the ammonia monooxygenase subunit A gene of complete ammonia-oxidizing Nitrospira
Source: Microbiol Spectr. 2024 Aug 21;12(10):e00516-24. doi: 10.1128/spectrum.00516-24 (PMC11448142; doi:10.1128/spectrum.00516-24)
Supplement: Supplemental material — Fig. S1 to S11; Tables S2 to S4 [file spectrum.00516-24-s0001.pdf]

Comprehensive evaluation of primer pairs targeting the ammonia monooxygenase subunit A gene of complete ammonia-oxidizing *Nitrospira*

Pieter Blom[1], Garrett J. Smith[1], Maartje A.H.J. van Kessel[1], Hanna Koch[1,2], Sebastian Lücker[1]

Supplemental Information

<u>This file contains:</u>

- Supplemental Figures S1 – S11
- Supplemental Tables S1 – S4

Supplemental Figures

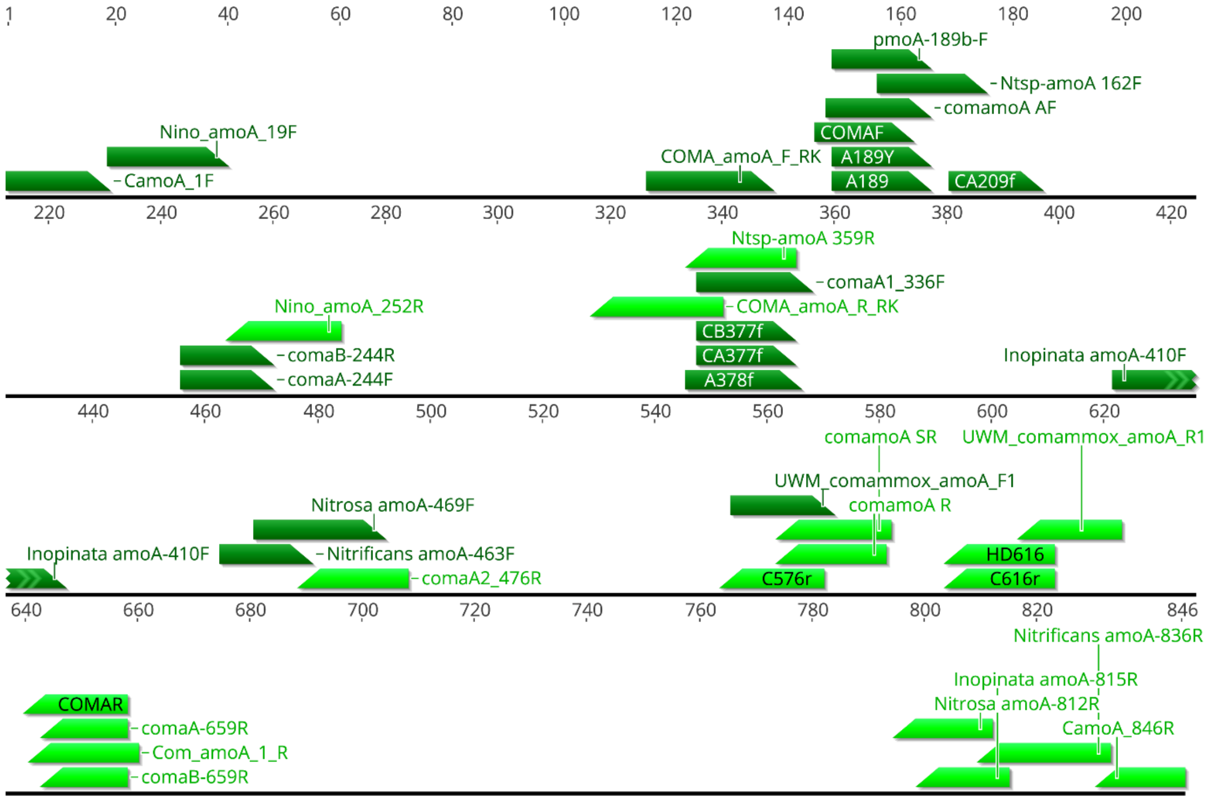

Supplemental Figure S1: Binding sites of comammox *Nitrospira amoA*-targeted primers. Shown are all published comammox *Nitrospira amoA* forward (dark green) and reverse primers (light green) and their binding location on a consensus comammox *amoA* gene sequence generated by aligning the sequences of *Nitrospira inopinata*, *Ca.* Nitrospira nitrificans, *Ca.* Nitrospira nitrosa, *Nitrospira* sp. AMP-1, and the clade B MAG *Nitrospira* sp. RCB.

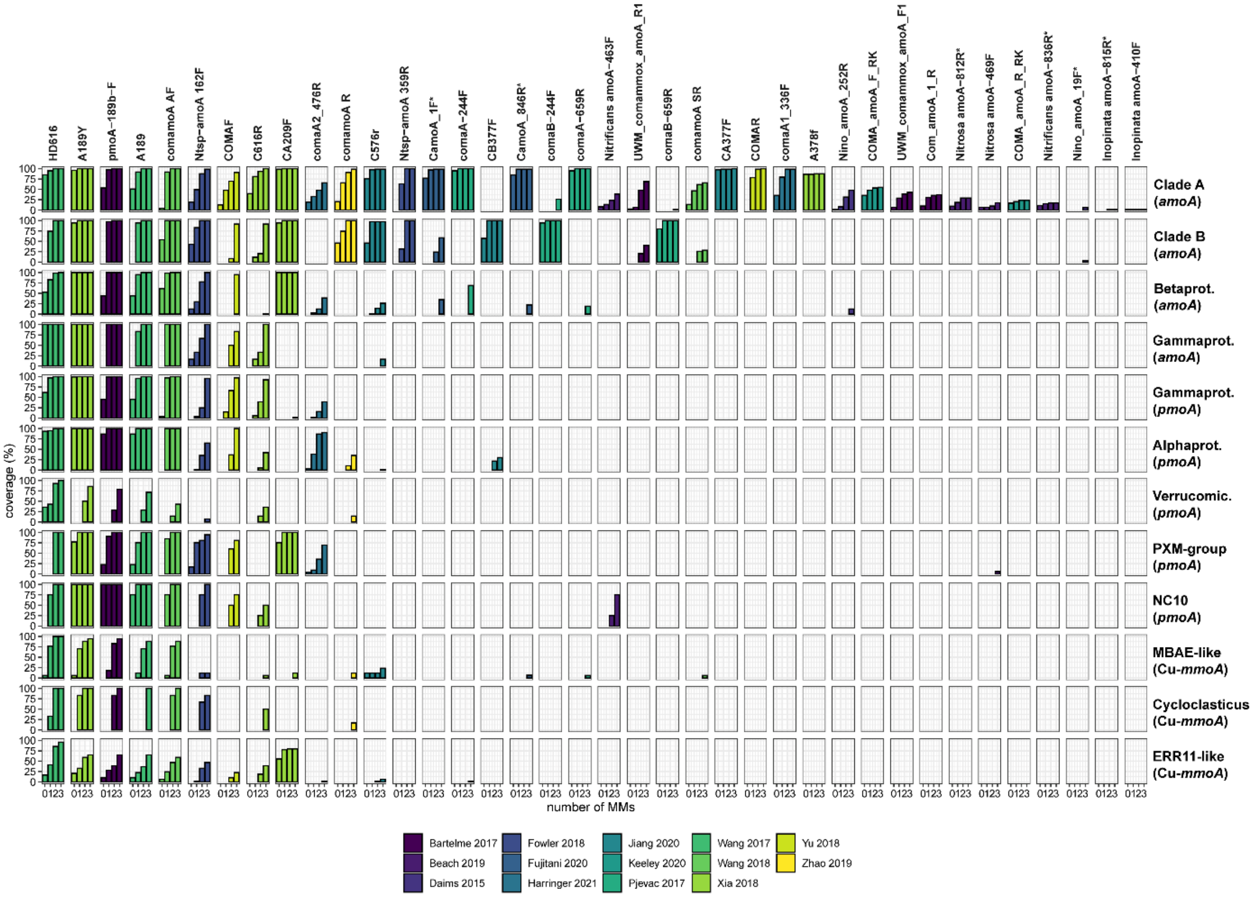

Supplemental Figure S2: Coverage and specificity of all comammox *Nitrospira amoA*-targeted primers. The percentage of sequences per group each primer matches to allowing for ≤ 3 MMs against their target sequences without MM at the 2 last positions of the 3’ end (i.e., coverage) is shown for clade A *amoA*, clade B *amoA*, betaproteobacterial (Betaprot.) *amoA,* gammaproteobacterial (Gammaprot.) *amoA,* gammaproteobacterial (Gammaprot.) *pmoA*, alphaproteobacterial (Alphaprot.) *pmoA,* Verrucomicrobial (Verrucomic.) *pmoA,* PXM-group *pmoA*, NC10 *pmoA*, MBAE-like Cu-*mmoA*, *Cycloclasticus* sp. Cu-*mmoA* and *Bradyrhizobium* sp. ERR11-like Cu-*mmoA* sequences. The results for each primer were colored based on its original publication. Primers analyzed using a smaller dataset comprising full-length sequences are marked with an asterisk.

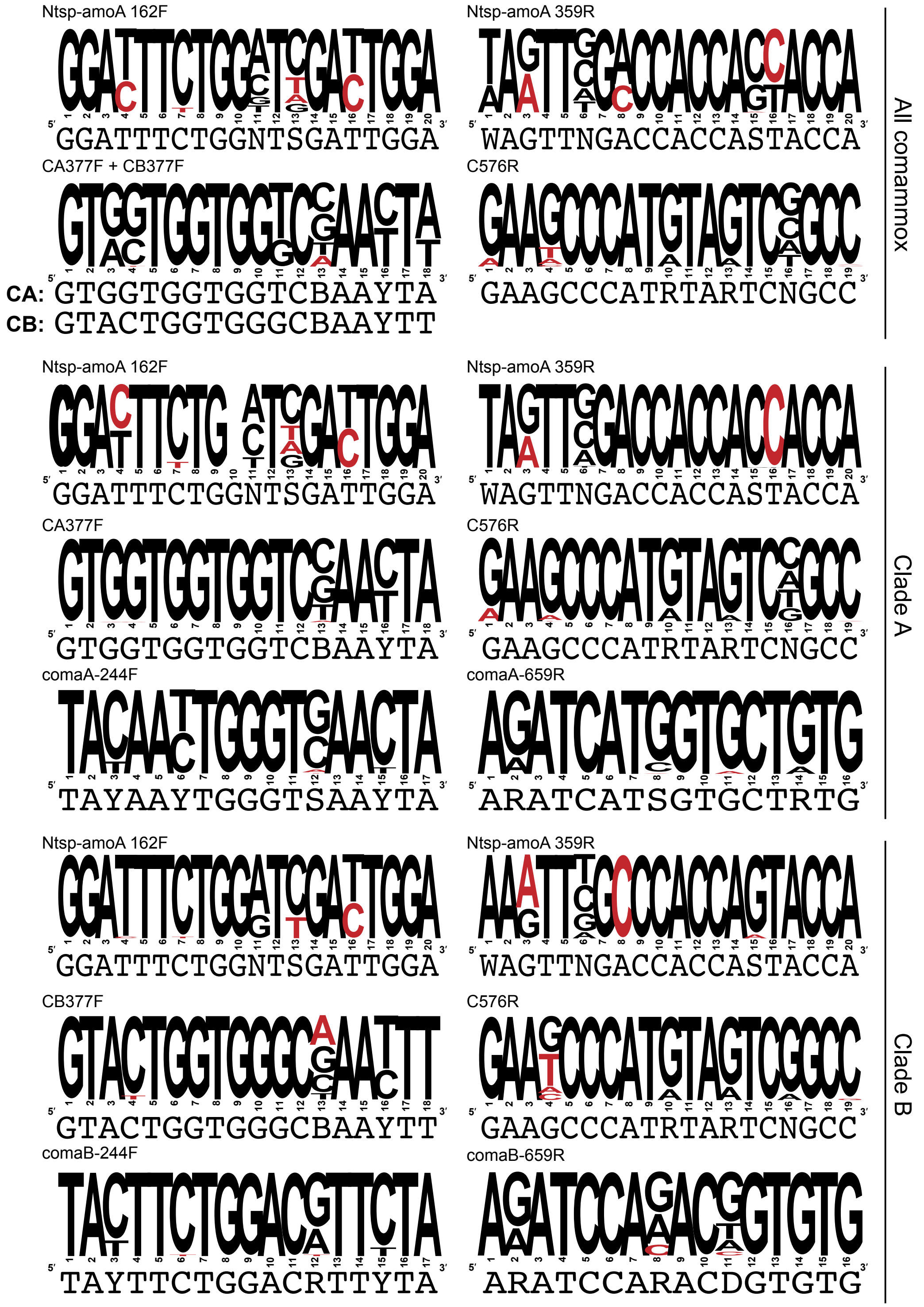

Supplemental Figure S3: Binding sites of selected primers on comammox *Nitrospira amoA* sequences. Primer sequences and the sequence logos of the alignment of all comammox (top), clade A comammox (middle), and clade B comammox (bottom) *amoA* sequences are shown. Nucleotides that do not match the primer are indicated in red. The sequence logos matching the reverse primers are shown as reverse complement sequences.

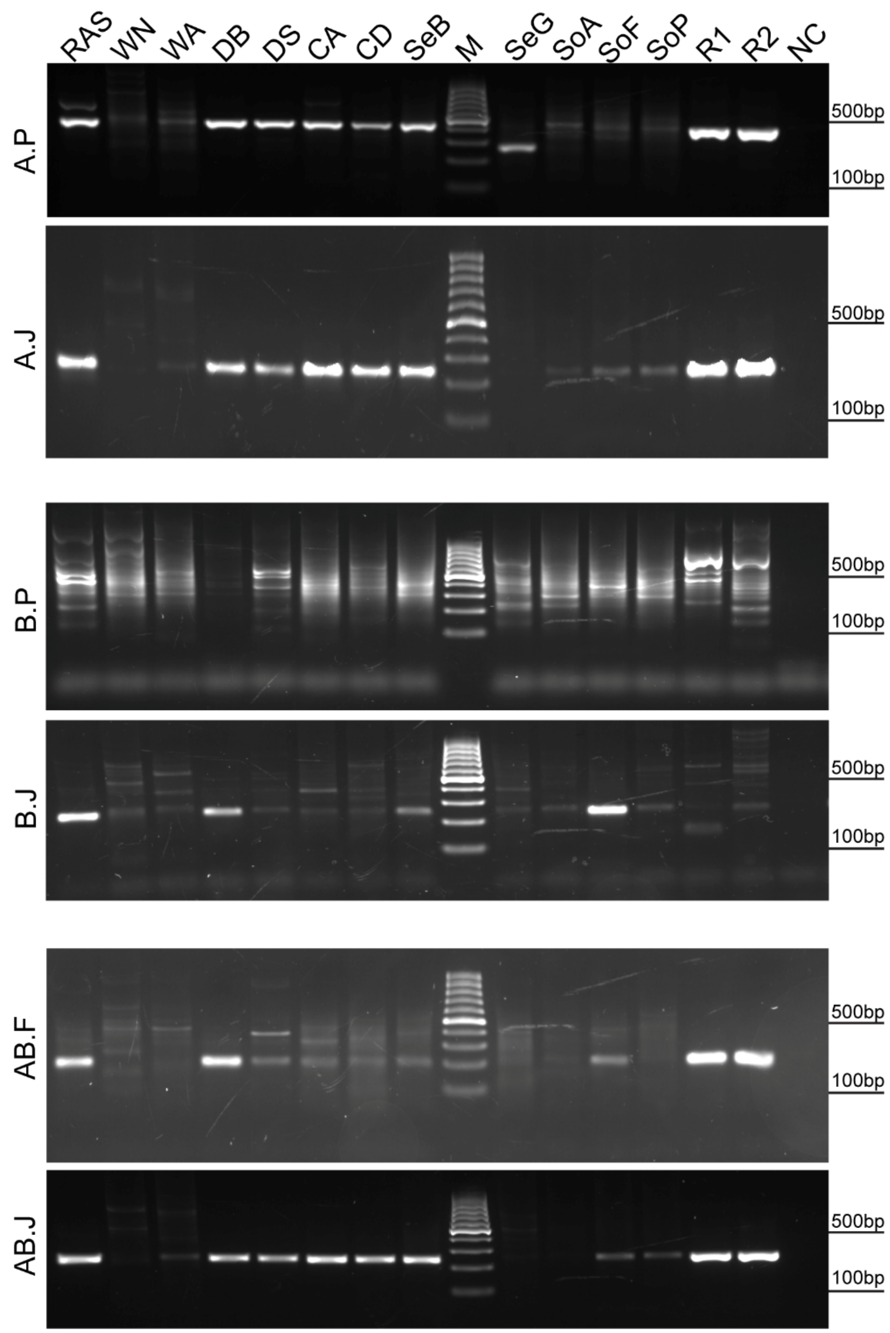

Supplemental Figure S4: Visualization of end-point PCR products obtained by applying selected primer pairs. Agarose gel electrophoresis of the end-point PCR products for all environments using comaA-244F/659R (A.P), CA377F/C576R (A.J), comaB-244F/659R (B.P), CB377F/C576R (B.J), Ntsp-amoA162F/359R (AB.F), and CA-CB377F/C576R (AB.J); NC, negative control; M, 100 bp marker. DNA extracts from the following habitats have been amplified: recirculating aquaculture system (RAS) biofilm, activated sludge samples from two different WWTPs (WA; WN), rapid sand filter samples from two different drinking water treatment plants (DB; DS), biofilm samples from canals in two Dutch cities (CA; CD), sediment samples from both freshwater (SeB) and brackish systems (SeG), soil samples from a cropland (SoA), a forest (SoF), and a park (SoP), and biomass from two nitrifying bioreactors (R1; R2).

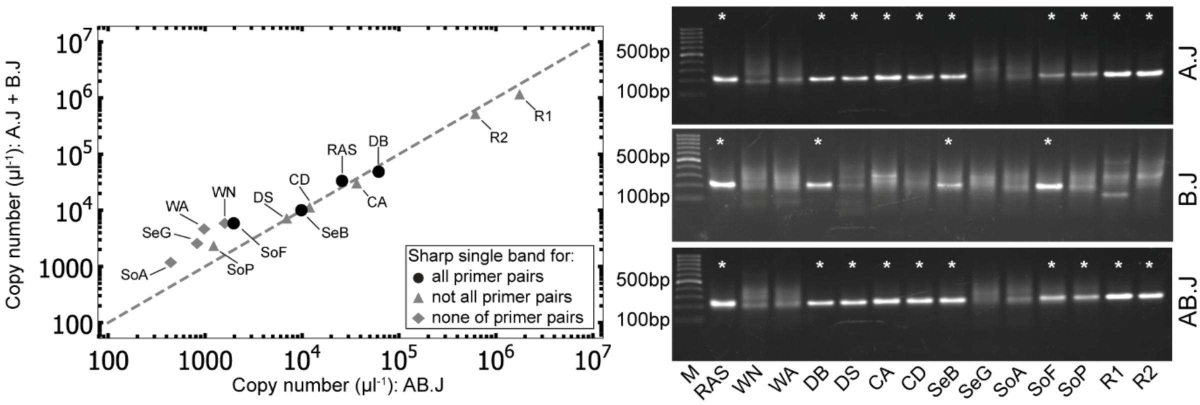

Supplemental Figure S5: Comparison of amplification quantity and quality using a mixture (CA-CB377F/C576R) or single forward primers (CA377F or CB377F) with C576R as reverse primer. (Left) Detected copy numbers for CA-CB377F/C576R (AB.J) against the sum of the copy numbers for CA377F/C576R (A.J) and CB377F/C576R (B.J). Black circles indicate a sharp band for all primer pairs, grey triangles indicate a defined band for CA-CB377F/C576R (AB.J) and grey diamonds indicate a lack of sharp bands for both primer pairs. (Right) Visualization of the obtained qPCR products by agarose gel electrophoresis. Sharp bands are indicated with asterisks; M, 100 bp marker. The agarose gels are also shown in **Figure 3**.

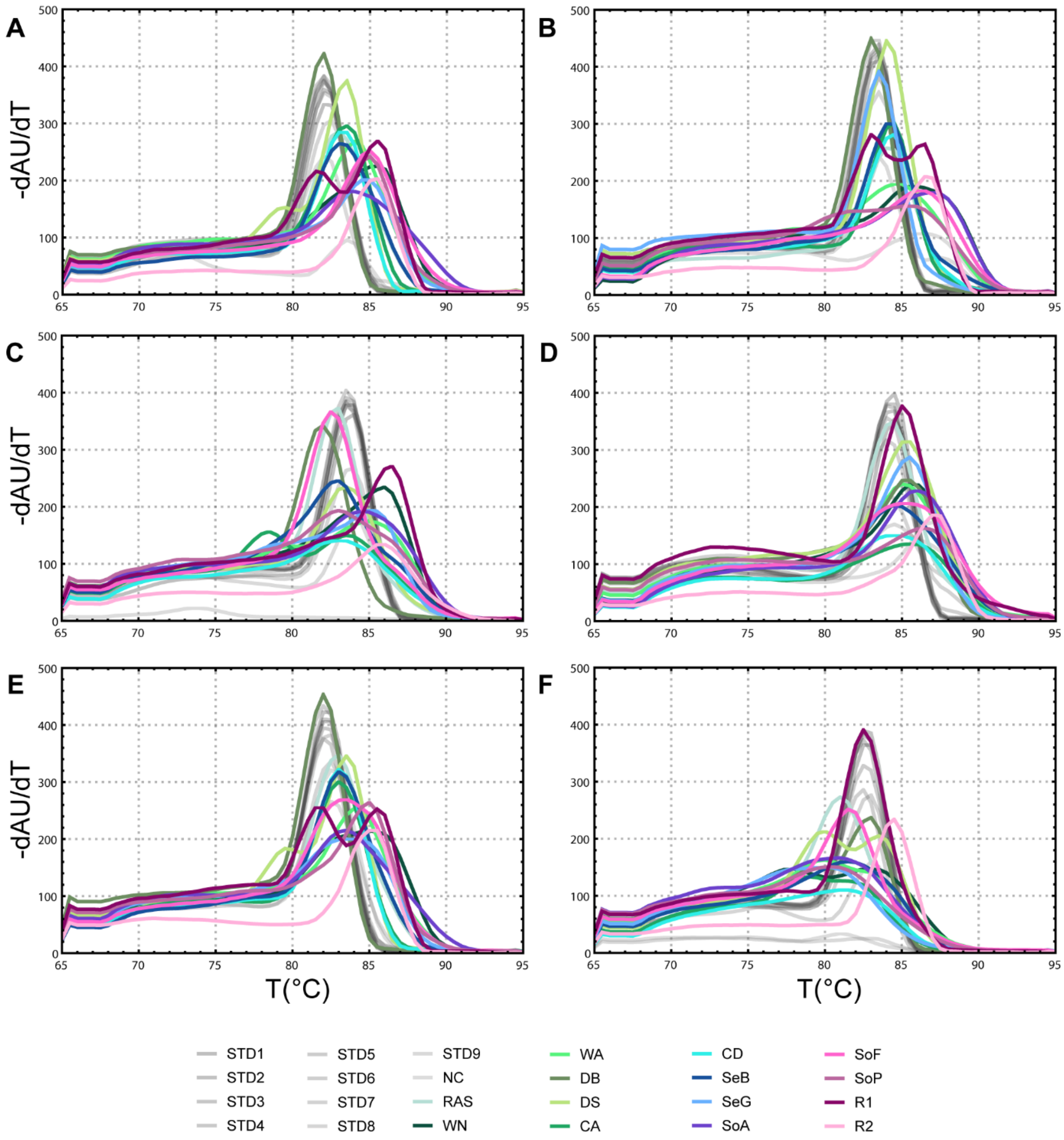

Supplemental Figure S6: Melting curves obtained after all qPCR assays. One of each triplicate melting curves is shown for each standard and sample using the primer pairs (A) CA377F/C576R (A.J), (B) CB377F/C576R (B.J), (C) CA-CB377F/C576R (AB.J), (D) comaA-244F/659R (A.P), (E) comaB-244F/659R (B.P), (F) Ntsp-amoA162F/359R (AB.F).

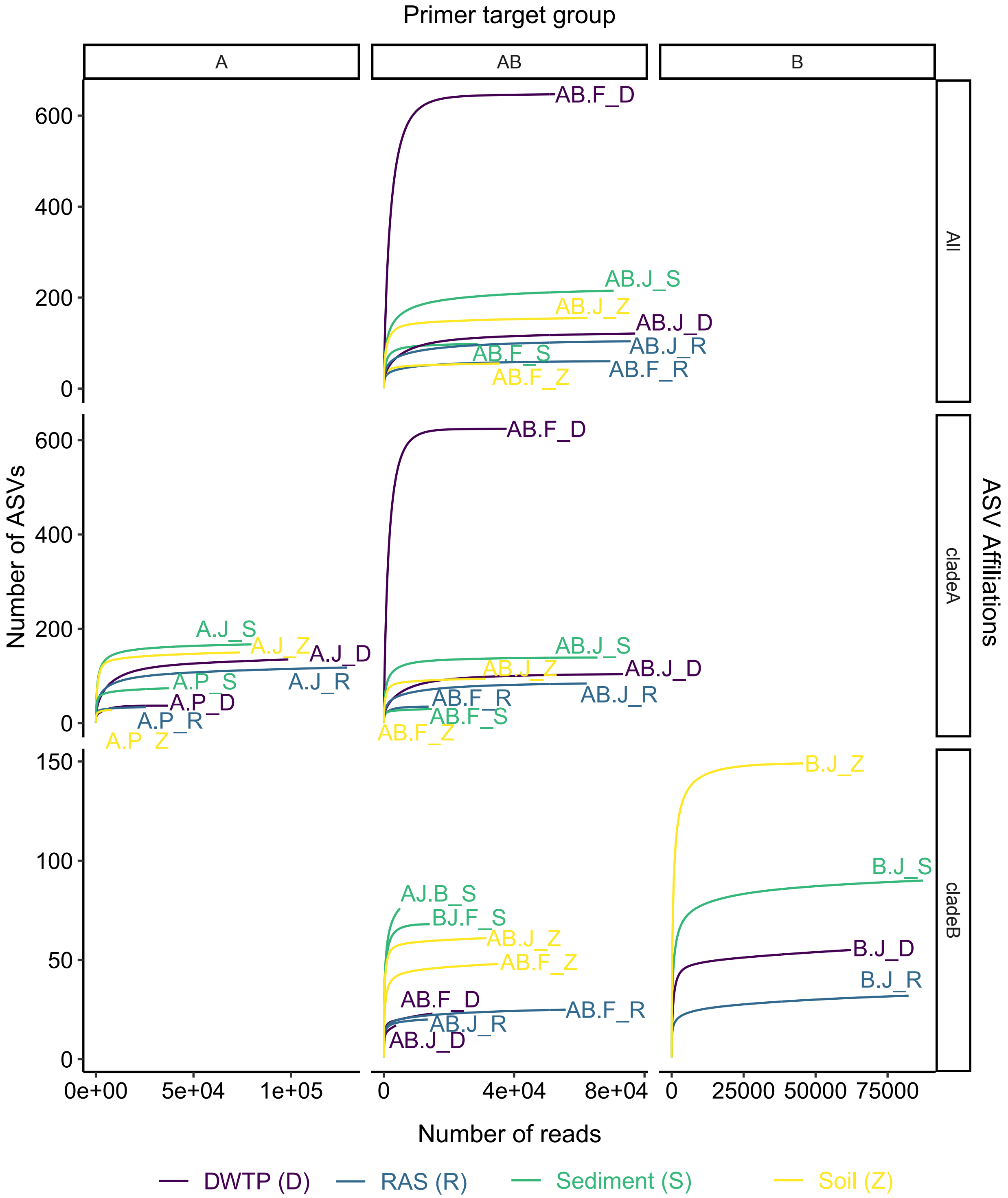

Supplemental Figure S7: Rarefaction curves for all comammox *Nitrospira amoA* amplicons. The number of ASVs versus the number of reads is shown for the target groups (horizontal) and ASV affiliations (vertical); abbreviations indicate primer pair (AB.F, Ntsp-amoA162F/359R; A.J, CA377F/C576R; B.J, CB377F/C576R; AB.J, CA-CB377F/C576R; A.P, comaA-244F/659R) and sample site (D, DB; R, RAS; S, SeB; Z, SoF).

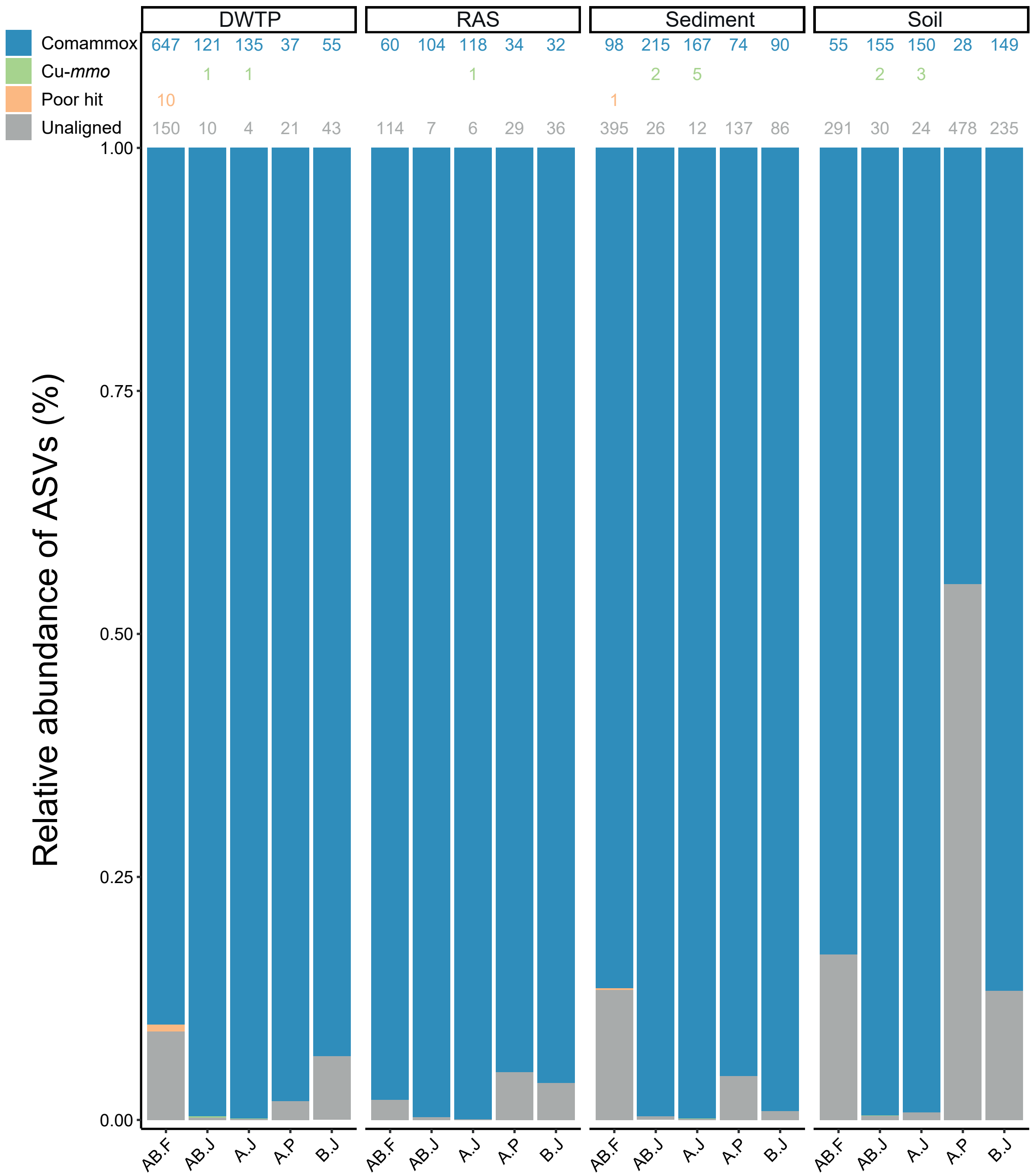

Supplemental Figure S8: Relative ASV abundance and absolute ASV counts for selected primer pairs and environments. The relative abundance of comammox *Nitrospira amoA*, other Cu-*mmoA*, poor hits, and unaligned ASVs (bottom) as well as the absolute number of ASVs (top) using the primer pairs Ntsp-amoA162/359R (AB.F), CA-CB377F/C576R (AB.J), CA377F/C576R (A.J), comaA-244F/659R (A.P), and CB377F/C576R (B.J) on DNA extracts from the DWTP (DB), RAS, sediment (SeB), and soil (SoF) samples.

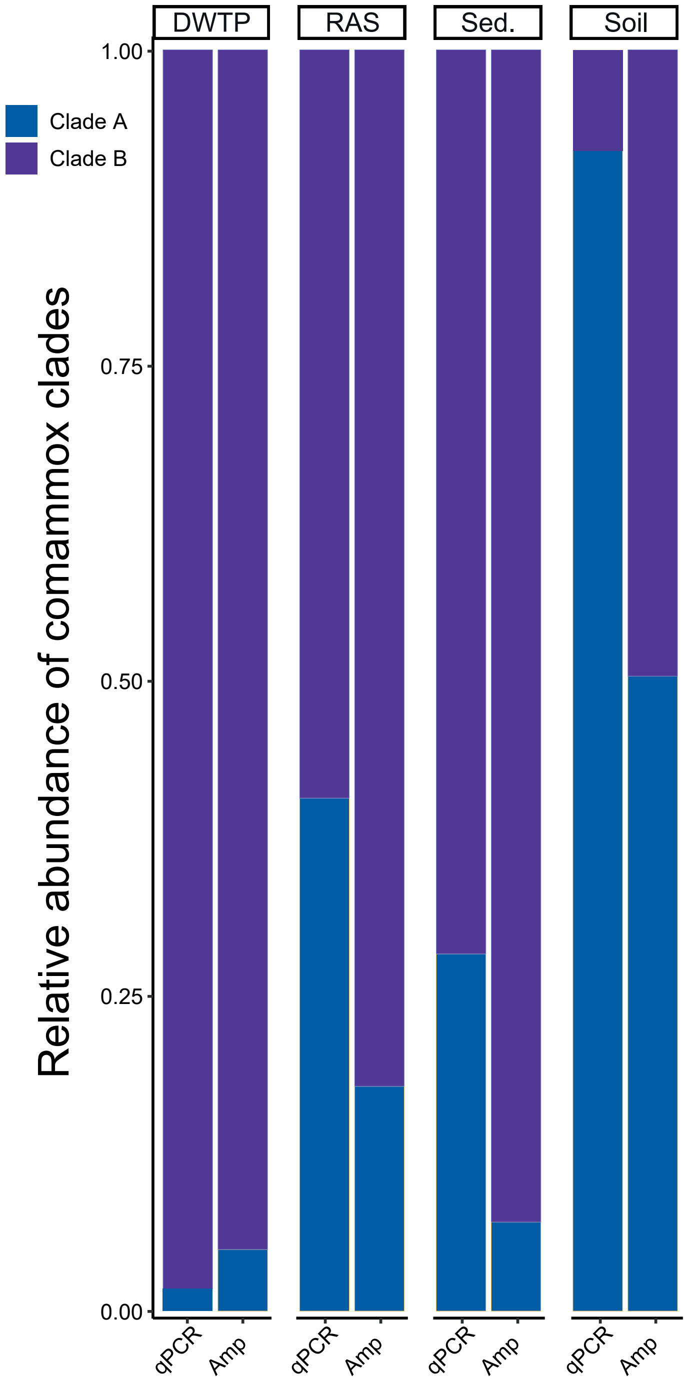

Supplemental Figure S9: Comparison of the ratio of clade A to clade B comammox *Nitrospira* using qPCR and amplicon sequencing. The relative abundance of each comammox clade is shown based on the results obtained from qPCR using the primer pairs CA377F/C576R (A.J) and CB377F/C576R (B.J) (left) and from amplicon sequencing (Amp) using CA-CB377F/C576R (AB.J) (right) on DNA extracts from the DWTP (DB), RAS, sediment (SeB), and soil (SoF) samples.

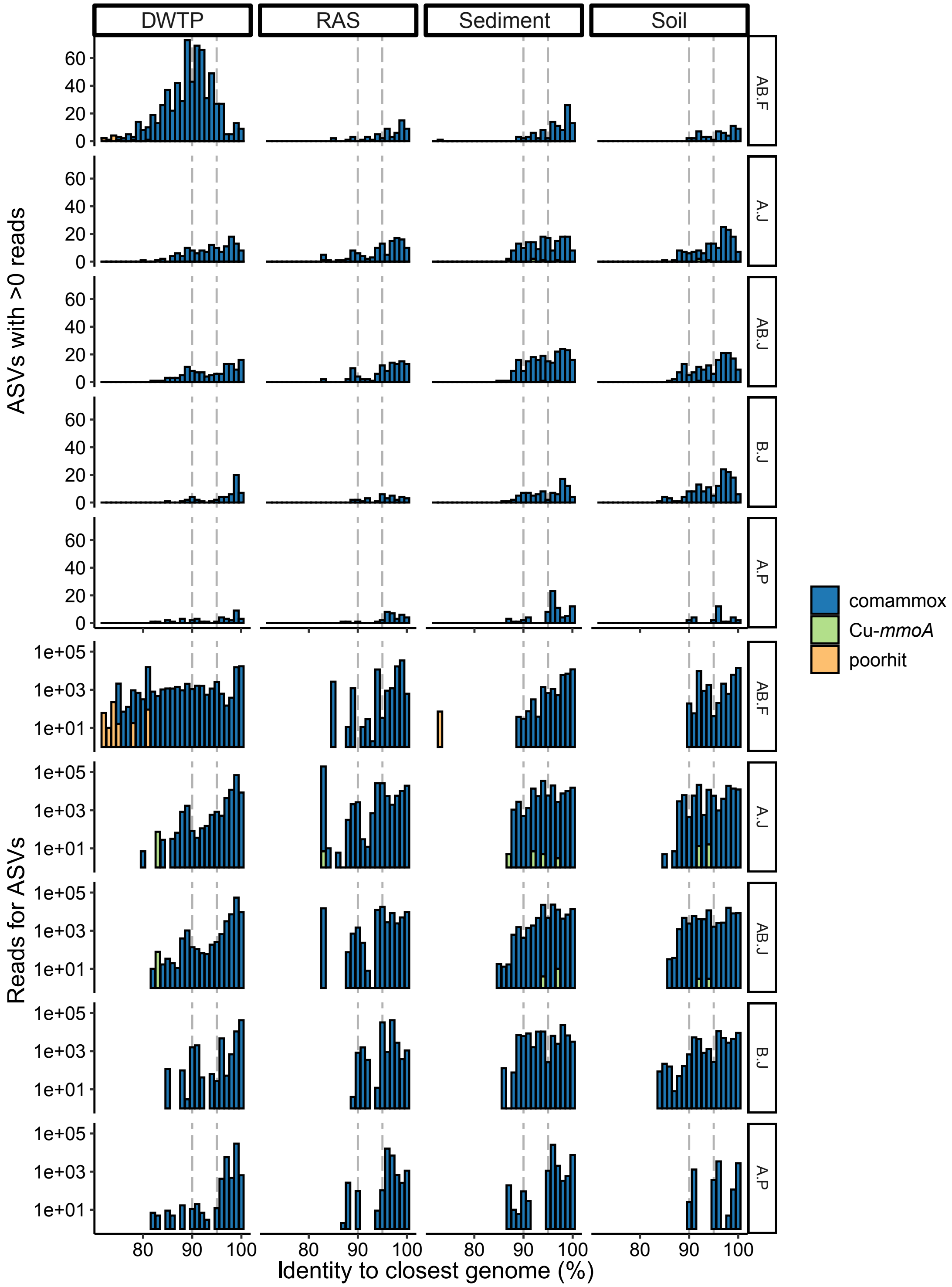

Supplemental Figure S10: ASV counts and number of reads compared to their identity to the closest reference sequence. Histograms of ASV counts (top) and read counts of ASVs (bottom) by their identity to the closest reference genome are shown for habitats DWTP (DB), RAS, sediment (SeB), and soil (SoF) and primer pairs Ntsp-amoA162/359R (AB.F), CA377F/C576R (A.J), CA-CB377F/C576R (AB.J), CB377F/C576R (B.J), and comaA-244F/659R (A.P). Comammox *Nitrospira amoA* hits are shown in blue, other Cu-*mmoA* hits in green, and poor hits in orange. Percentages of 90% and 95% are indicated with dashed grey lines.

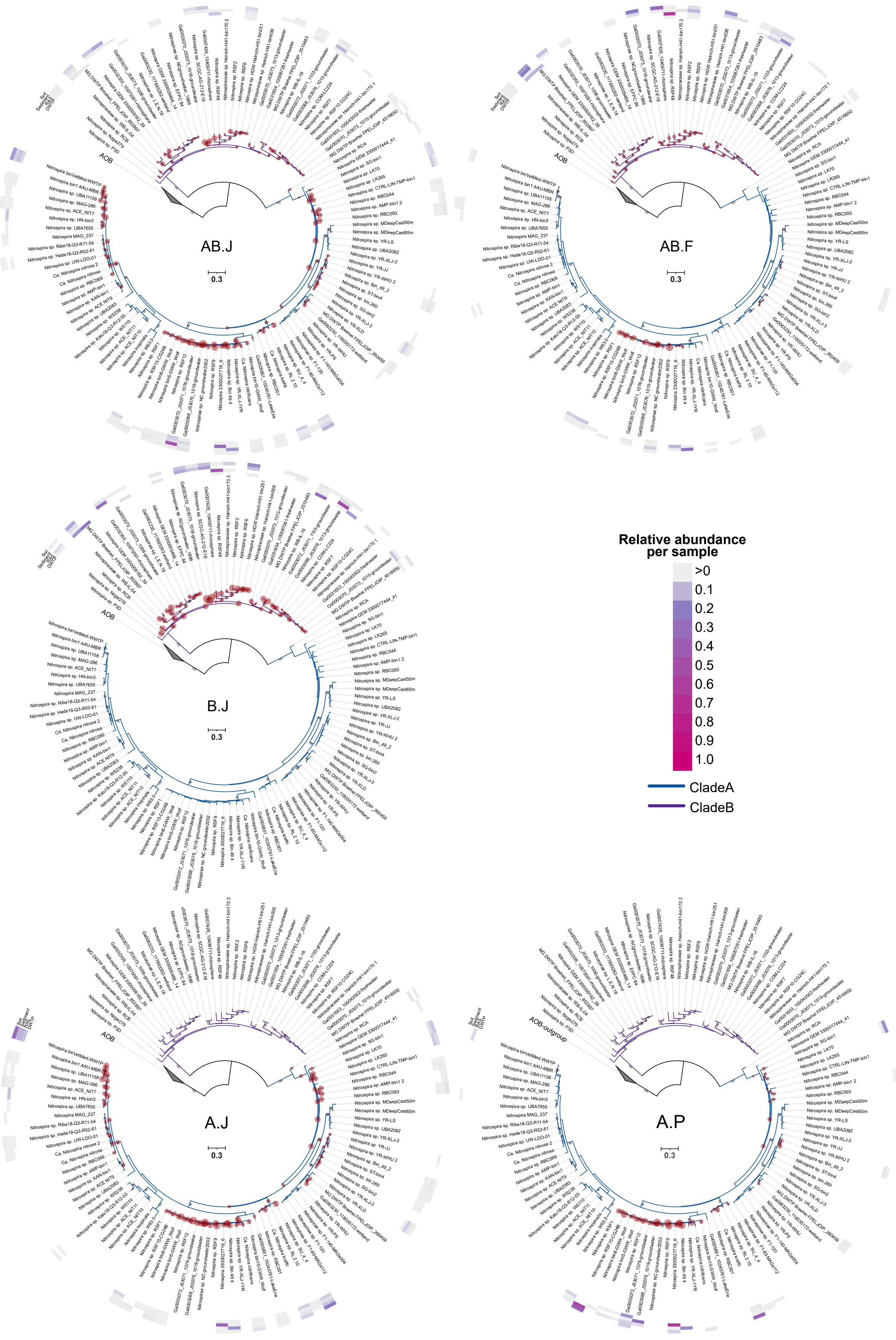
AB.J
AB.F
B.J
A.J
A.P
0.3
AOB
AOB-outgroup
Soil
Sediment
WWTP
DWTP
Relative abundance per sample
>0
0.1
0.2
0.3
0.4
0.5
0.6
0.7
0.8
0.9
1.0
CladeA
CladeB

Supplemental Figure S11: Phylogenetic placement of ASVs obtained with selected primer pairs. Shown are maximum likelihood trees of comammox *Nitrospira amoA* gene sequences aligned using Muscle and calculated using RAxML-NG with 500 replications and the TIM3+F+I+G4 model. The number of ASVs that were placed onto the topology of the tree using EPA-NG is indicated by the size of the red circles. The heatmaps in the outer rings of the trees indicate the relative abundance of the ASVs matching this genomic reference sequence within an environment (from inner to outer ring: DWTP (DB), RAS, sediment (SeB), soil (SoF)). Bootstrap support ≥ 90% is indicated. 6 *Nitrosomonadaceae amoA* sequences were used as outgroup.

Supplemental Tables

Supplemental Table S1 (provided as separate Excel file): Overview of all primer characteristics. Primer names are given along with the binding position on the *Nitrospira inopinata* and clade-specific reference *amoA* sequence, the direction of primers, their GC-content, number of mismatches towards reference sequence, original publication, and their sequence.

Supplemental Table S2: qPCR statistics for each run. The efficiency (eff.), r^2, slope, and intercept of each standard curve are given for all primer pairs tested. Abbr.; abbreviation.

Primer pair	Abbr.	Eff. (%)	r^2	Slope	Intercept
**CA377F/C576R**	A.J	93.9	0.996	-3.476	38.369
**CB377F/C576R**	B.J	85.7	0.999	-3.719	41.197
**CA-CB377F/C576R**	AB.J	92.7	0.986	-3.510	40.268
**comaA-244F/659R**	A.P	93.7	0.986	-3.482	38.767
**comaB-244F/659R**	B.P	98.4	0.997	-3.362	38.740
**Ntsp-amoA162/359R**	AB.F	100.8	0.980	-3.303	38.836

Supplemental Table S3: Amplicon sequencing details. Read and base pair counts reflect those assigned to an amplicon sequence variant from any environmental sample.

Primer F/R	Target clade	Primer F/R length	Read F/R length	Overlap length	Total reads	Total bps
**Ntsp-amoA162F/359R**	A, B	20/20	160/160	160	610,814	85,513,960
**CA377F/C576R**	A	18/19	208/209	130	897,214	170,470,660
**CB377F/C576R**	B	18/19	208/209	130	692,744	131,621,360
**CA-CB377F/ C576R**	A, B	18/19	208/209	130	668,454	127,006,260
**comaA-244F/ 659R**	A	17/16	277/276	12	331,324	86,144,240

Supplemental Table S4: Classification of all amplicon sequencing reads. Reads were categorized as AOB *amoA*, clade A *amoA*, clade B *amoA*, poor hit Cu-*mmoA*, or without hit (unknown), and sorted by primer pair and sample; relative abundances are shown as percentage.

Primer pair	Sample	Total ASV Counts	AOB (%)	Clade A (%)	Clade B (%)	Poor hit (%)	Unknown (%)
**Ntsp-amoA 162F/359R**	DB	807	-	64.6	25.6	0.73	9.1
	RAS	174	-	19.4	78.6	-	2.0
	SeB	494	-	44.3	42.2	0.22	13.3
	SoF	346	-	0.86	82.1	-	17.0
**CA377F/ C576R**	DB	140	0.076	99.8	-	-	0.079
	RAS	125	0.005	99.9	-	-	0.042
	SeB	184	0.025	99.9	-	-	0.089
	SoF	177	0.039	99.2	-	-	0.74
**CA-CB377F/ C576R**	DB	132	0.10	94.8	4.9	-	0.24
	RAS	111	-	82.0	17.8	-	0.24
	SeB	243	0.020	92.6	7.0	-	0.35
	SoF	187	0.010	49.5	50.1	-	0.41
**CB377F/ C576R**	DB	98	-	-	93.5	-	6.5
	RAS	68	-	-	96.2	-	3.8
	SeB	176	-	-	99.1	-	0.90
	SoF	384	-	-	86.7	-	13.3
**comaA244F/ 659R**	DB	58	-	98.1	-	-	1.9
	RAS	63	-	95.1	-	-	4.9
	SeB	211	-	95.5	-	-	4.5
	SoF	506	-	44.9	-	-	55.1
